# Supplementary material for: An allosteric inhibitor of the Zika virus NS2B-NS3 protease with oral efficacy in mouse models
Source: Nat Commun. 2026 Feb 10;17:1439. doi: 10.1038/s41467-026-68943-x (PMC12891677; doi:10.1038/s41467-026-68943-x)
Supplement: Supplementary file 1 — Supplementary Information [file 41467_2026_68943_MOESM1_ESM.pdf]

Supplementary Information

An allosteric inhibitor of the Zika virus NS2B-NS3 protease with oral efficacy in mouse models

Jesus M. Ontoria[1]*✉, Esther Torrente[1], Antonino Missineo[2], Cristina Alli[2], Rita Graziani[2], Silvia Conti[2], Monica Bisbocci[2], Antonio Quotadamo[1], Federica Ferrigno[1], Alessandra Corio[1], Giovanni Ievoli[1], Leda Bencheva[1,4], Jérôme Amaudrut[1], Silvana Vasile[1], Elisa Beghetto[2], Chantal Paolini[2], Nadine Alaimo[2], Maria Veneziano[1], Martina Nibbio[1], Maria V. Orsale[1], Giulia Proto[1], Fabrizio Colaceci[1], Laura Orsatti[1], Vincenzo Pucci[1,5], Romano Di Fabio[1,6], Licia Tomei[2], Christian Montalbetti[1], Alberto Bresciani[2,7], Carlo Toniatti[3], Giacomo Paonessa[2]*✉

*✉Corresponding authors: j.ontoria@irbm.com, g.paonessa@irbm.com

Authors Affiliations:
[1]Department of Drug Discovery, IRBM S.p.A., 00071, Pomezia, Italy
[2]Department of Biology and Translational Research, IRBM S.p.A., 00071, Pomezia, Italy
[3]Chief Scientific Officer, IRBM S.p.A., 00071, Pomezia, Italy
[4]Present Address: Sibylla Biotech S.p.A, Via Lillo del Duca, 10, 20091 Bresso, Italy
[5]Present Address: Johnson & Johnson, Turnhoutseweg 30, 2340 Beerse, Belgium
[6]Present Address: Drug Discovery Unit, Vita Salute San Raffaele University, Via Olgettina, 58, 20132 Milan, Italy
[7]Present Address: Tycho S.r.l., Via Donini 20, 24129 Bergamo. Italy.

Table of contents:

SI Figure 1 |	HTS assay performance and EC_{50} determination for mycophenolic acid and NITD008	S3-4
SI Figure 2 |	HTS results and Hit triage workflow	S5-6
SI Table 1 |	Small molecule screening data	S7-8
SI Figure 3 |	Replicon cDNA sequence for the NS3 coding region	S9
SI Figure 4 |	ZIKV replicon nucleotide sequence and annotated features	S10-12
SI Figure 5 |	Multiple sequence alignment of orthoflavivirus NS2B and NS3 proteins	S13
SI Figure 6 |	Docking pose of the *S*-(-)-IRBM-Z-1 enantiomer in complex with the ZIKV NS2B-NS3 protease	S14
SI Table 2 |	Biological and ADME data comparison of known ZIKV NS2B-NS3 allosteric inhibitors	S15
SI Figure 7 |	Comparison of *R*-(+)-IRBM-Z-1 and compound 9 binding sites.	S16
SI Figure 8 |	Comparison of *R*-(+)-IRBM-Z-1 and compound 12R binding sites	S17
SI Figure 9 |	Comparison of *R*-(+)-IRBM-Z-1 and compound 1 binding sites	S18
SI Table 3 |	Data collection and refinement statistics (anisotropic statistics, molecular replacement)[1] for the ZIKV protease and *R*-(+)-IRBM-Z-	S19

	1 complex (PDB ID 9TPG) and the for the ZIKV protease and IRBM-Z-2 complex (PDB ID 9IBY)	
SI Figure 10 |	Superposition of the ZIKV NS2B-NS3 - *R*-(+)-IRBM-Z-1 complex with the apo ZIKV NS2B-NS3 closed conformation	S20
SI Figure11 |	Crystal structure of ZIKV NS2B-NS3 in complex with IRBM-Z-2.	S21
SI Figure 12 |	Biophysical characterization of IRBM-Z-1 and IRBM-Z-2	S22
SI Figure 13 |	ZIKV infection assay of IRBM-Z-2 in HuH-7 cells.	S23
SI Table 4 |	Hepatocyte stability of IRBM-Z-2	S24
SI Figure 14 |	*In vivo* pharmacokinetic profile of IRBM-Z-2 after dosing at 100 mpk (PO and IP)	S25
SI Figure 15 |	IP and PO repeated administration of compound IRBM-Z-2 at 100 mg/kg, once a day for 5 days	S26
SI Figure 16|	Pharmacokinetic profile of compound IRBM-Z-2 after repeated oral administration to C57BL/6 mice at 100, 200 and 300 mg/kg, once a day for 7 days	S27
SI Figure 17 |	PK profile of IRBM-Z-2 after repeated oral administration to C57BL/6 mice at 100 mg/kg BID	S28
SI Table 5 |	IP 5-Day PoC study design	S29
SI Table 6 |	PO 14-Day Survival and efficacy study design	S29
SI Figure 18 |	Preclinical safety panel for IRBM-Z-2	S30-32
Supplementary Methods		S33
	Kinetic solubility determination	S33
	PAMPA permeability assay	S33
	MDCK permeability assay	S33
	Microsomal stability intrinsic clearance determination	S34
	Hepatocyte stability studies	S35
	In Vitro CYP inhibition assay	S35
	In vitro human hepatocyte CYP induction assay	S36
	hERG polarization assay	S36
	Plasma protein binding determination	S36
	Synthesis of IRBM-Z-1, *R*-(+)-IRBM-Z-1 and IRBM-Z-2	S38-42
	^{1}H NMR, ^{19}F NMR and ^{13}C NMR spectra and UPLC-MS of compounds IRBM-Z-1, and IRBM-Z-2	S43-54
Supplementary References		S55

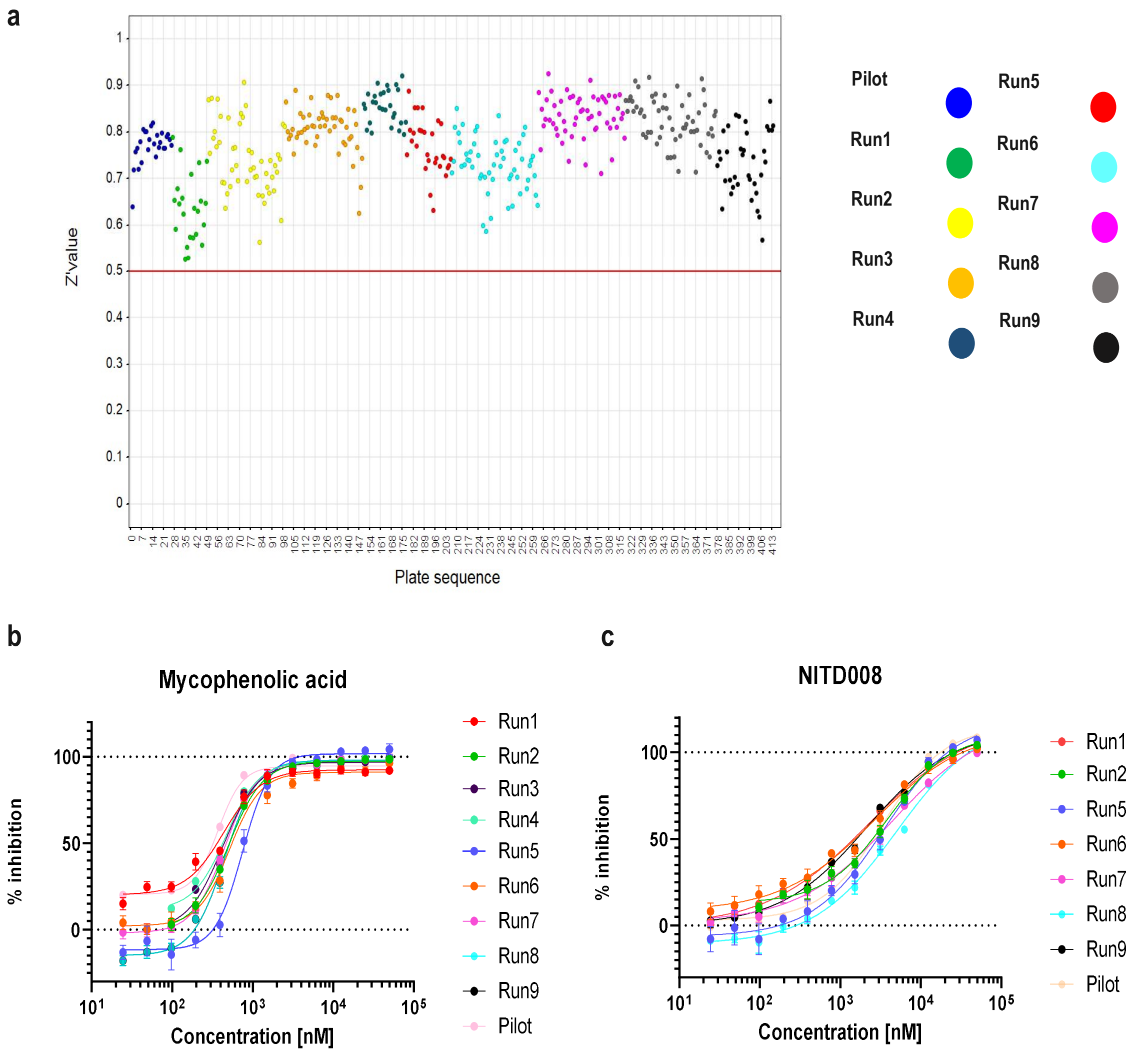

d

	Mycophenolic acid			NITD008		
	Mean EC50 nM	std	n	Mean EC50 nM	std	n
Pilot	373		1	3436		1
Run1	440	20	4	2238		1
Run2	497	33	4	3934	356	4
Run3	425	20	3	NT		
Run4	467		1	NT		
Run5	746	18	4	3684	190	3
Run6	509	44	4	2386	283	4
Run7	448	19	4	3950	696	4
Run8	450	24	4	3299	154	4
Run9	277	25	4	2105	176	3

Supplementary Figure 1 | HTS assay performance and EC_{50} determination for mycophenolic acid and NITD008. a Z' value obtained for each of the 414 plates screened grouped by run: pilot (blue), run 1 (green), run 2 (yellow), run 3 (orange), run 4 (dark green), run 5 (red), run 6 (light blue), run 7 (purple), run 8 (grey) and run 9 (black). The red line indicates the minimum acceptable Z' threshold value. **b** and

c Dose-response curve and EC_{50} determination for mycophenolic acid and NITD008, respectively. In both cases, percent inhibition of enzymatic activity is plotted against compound concentration (nM). EC_{50} values were calculated using Prism software. **d** Summary table showing the EC_{50} values for mycophenolic acid and NITD008 (mean, standard deviation and number of biological repeats). NT: not tested.

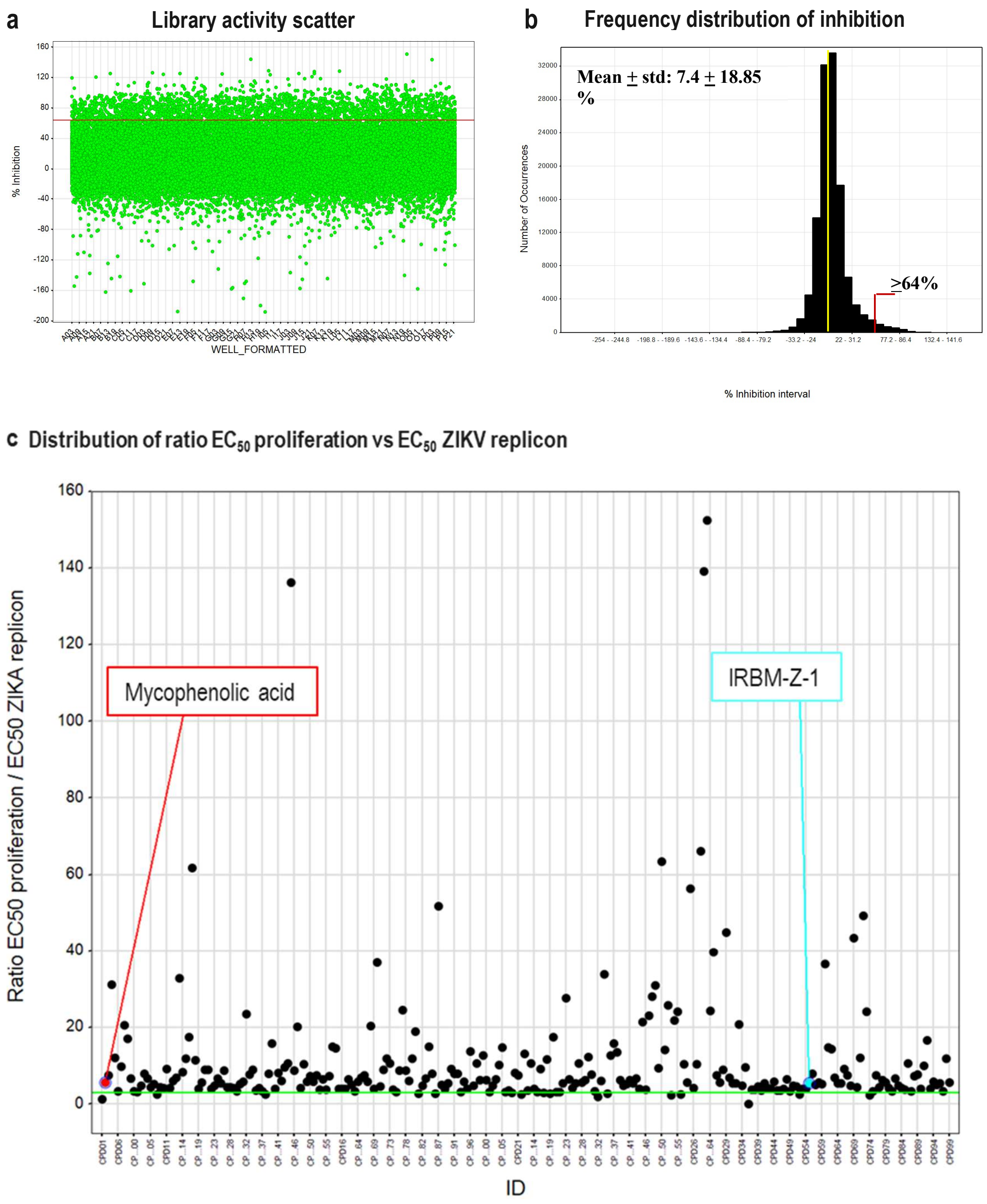

Supplementary Figure 2 | HTS results and Hit triage workflow. Compounds were considered as hits if they exhibited > 64% inhibition at 10 µM, corresponding to three standard deviations above the mean inhibition value of 7.4% (see panel a and b). This threshold yielded 2,210 primary hits, corresponding to a hit rate of 1.8%. Compounds were further filtered based on the following criteria: purity > 80%, molecular weight < 600 Dalton, HBA > 8, HBD < 5, tPSA < 130, logP between 0 and 5, exclusion of PAINS motifs[1-5-40], or having undesirable chemical structures. This selection narrowed the list to 985 compounds for reconfirmation. These compounds were subsequently tested at two concentrations (10 and 1 µM) in the ZIKV replicon assay and in a Vero cell proliferation assay, to flag cytotoxic candidates. This screening step yielded 699 compounds confirmed as hits that were taken forward for EC_{50} determination. Among them, 266 compounds showed EC_{50} values below 7.5 µM in the replicon assay and an EC_{50} ratio higher than 3 in the proliferation/replicon assay. Selected

compounds from this group were used to generate compound-resistant ZIKV replicon cell lines. **a** Scatter plot of library activity. Red line, the cut-off limit chosen to identify the hits. **b** Frequency distribution of percent inhibition across the 120,000-compound library. In yellow: distribution peak and in red the mean + 3σ cutoff, corresponding to approximately 64% inhibition. The slightly positive mean value indicated an uneven Gaussian distribution registered during the full HTS campaign which was due to a positioning effect on the control wells. **c** Distribution of EC_{50} ratios between the proliferation and ZIKV replicon assays. In light blue, IRBM-Z-1, in red Mycophenolic acid. A subset of 120 compounds with ratio >3 was selected for follow-up.

Category	Parameter	Description
Assay	Type of assay	Cell-based assay
	Target	Zika replication using replicon (for the ZIKV subgenomic replicon construction, we used the sequence of a clone of the Natal RGN isolate of Asian lineage (GenBank: KU527068.1) with the modification reported in the material and method
	Primary measurement	Detection of NanoLuc reporter
	Key reagents	Nano-Glo reagent (Promega, Cat. #N1150)
	Assay protocol	As reported in Replicon inhibition and cell viability assays session in material and method
	Additional comments	
Library	Library size	120,000-compound library
	Library composition	The composition of the library reflects high structural diversity, incorporating several chemotypes and structural classes
	Source	
	Additional comments	Molecular Properties: MW= 380, LogD= 2.3, LogP = 2.5, HBA= 6, HBD= 1, Rotable Bonds= 5, TPSA= 68, CNS-MPO= 5, QED = 0.7
Screen	Format	384-well plates
	Concentration(s) tested	10 μM, 0.5% DMSO
	Plate controls	In each plate, 16 positive control wells received mycophenolic acid (50 μM, from Sigma-Aldrich, M3536) and 16 negative control wells received 0.5% DMSO.<br>Dose-response curve determination for mycophenolic acid and NITD008 for each run
	Reagent/ compound dispensing system	Compounds were dispensed via acoustic liquid handler (ATS-100 EDC or Echo650, Beckman); cells, Nano-Glo and Cell-Titer-Glo reagents were dispensed using Tempest (Formulatrix)
	Detection instrument and software	Envision reader (PerkinElmer); Software version 1.13.3009.1401
	Assay validation/QC	Z' value > 0.5 for each plate; Dose-response curve determination for mycophenolic acid and NITD008 for each run in the expected range
	Correction factors	Automatically removal of outliers from the positive and negative control wells based on the median (Dotmatics, Study software used for the data analysis- Dotmatics-Bioshops Stortford, UK, Version 5.0). On the normalized HTS data, a plate correction was applied for each run (Plate correction is a script in Vortex software, based on median. Vortex as apert of Dotmatics, Bioshops Stortford, UK, Vortex 6.1.2.1372-s)
	Normalization	Data were analyzed as normalized between 0 and 100% inhibition (Negative control wells and positive control well).<br>For EC_{50} determinations, the dose-response was fitted with a 4-p logistic regression approach.
	Additional comments	Graphs reported in the supplementary information (Supplementary Figures 1 and 2)
Post-HTS analysis	Hit criteria	Compounds were considered as hits if they exhibited > 64% inhibition at 10 μM, corresponding to three standard deviations above the mean inhibition value of 7.4%. 2,210 primary hits were further filtered based on the following criteria: purity > 80%, molecular weight < 600 Dalton, HBA > 8, HBD < 5, tPSA < 130, logP between 0 and 5, exclusion of PAINS motifs1-5-40, or having undesirable chemical structures. This selection narrowed the list to 985 compounds for reconfirmation. This screening step yielded 699 compounds confirmed as hits that were taken forward for EC_{50} determination. Among them, 266 compounds showed EC_{50} values below 7.5 μM

	in the replicon assay and an EC_{50} ratio higher than 3 in the proliferation/replicon assay
Hit rate	2,210 primary hits, corresponding to a hit rate of 1.8%
Additional assay(s)	Cell viability assessed using CellTiter-Glo (Promega, Cat. #G7573)
Confirmation of hit purity and structure	Inclusion criteria require compliance with high purity standards, with UV-UPLC mass purity greater than 90%, assessed using a standard analytical method. UPLC-MS analysis was conducted on a Waters UPLC system with both Diode Array detection and Electrospray (+'ve and -'ve ion) MS detection. The mobile phase comprised H2O containing 0.1% formic acid (A) and MeCN containing 0.1% formic acid (B) in the following linear gradient: 90% A (0.1 min), 90%-0% A (2.5 min), 0% A (0.3 min), 0-90% A (0.1 min) with a flow rate 0.5 mL/min. The stationary phase was a Waters Acquity UPLC BEH C18 1.7um (2.1x50mm) column, 130A.
Additional comments	

Supplementary Table 1 | Small molecule screening data.

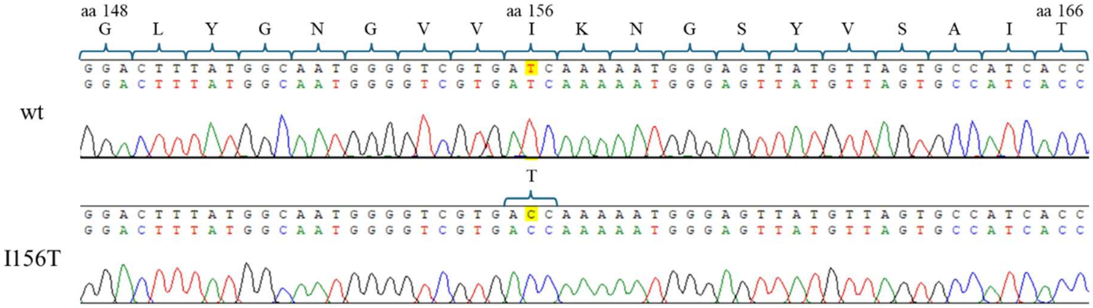

Supplementary Figure 3 | Replicon cDNA sequence for the NS3 coding region. Representative Sanger sequencing chromatograms showing the replicon cDNA sequence corresponding to the NS3 coding region (amino acids 148-166) from the ZIKV wild-type stable replicon and an IRBM-Z-1-resistant clone harboring the I156T mutation. The position of the I156T substitution is indicated, with the corresponding nucleotide change clearly visible in the chromatograms.

a

agttgttgatctgtgtgaatcagactgcgacagttcgagtttgaagcgaaagctagcaacagtatcaacaggttttattttggatttggaaacgagagtt
tctggtcatgaaaaacccaaaaaagaaatccggaggattccggattgtcaatatgctaaaacgcggagtagcccgtgtgagcccctttgggggctt
gaagatgtggttgggtctgaacacaaagaatggatctatttcccttatgtgcttggccttagggggagtgttgatcttcttatccacagccgtctctgctg
atgtggggtgctcggtggacttctcaaagaaggagacgagatgcggtacaggggtgttcgtctataacgacgttgaagcctggagggacaggtac
aagtaccatcctgactccccccgtagattggcagcagcagtcaagcaagcctgggaagatggtatctgcgggatctcctctgtttcaagaatggaga
acatcatgtggagatcagtagaaggggagctcaacgcaatcttggaagagaatggagttcaactgacggtcgttgtgggatctgtaaaaaacccca
tgtggagaggtccacagagattgcccgtgcctgtgaacgagctgccccacggctggaaggcttgggggaaatcgtacttcgtcagagcagcaaa
gacaaataacagctttgtcgtggatggtgacacactgaaggaatgcccactcgaacatagagcatggaacagctttcttgtggaggatcatgggttc
ggggtatttcacactagtgtctggctcaaggttagagaagattattcattagagtgtgatccagccgttattggaacagctgttaaggggaaggaggc
tgtacacagtgatctaggctactggattgagagtgagaagaatgacacatggaggctgaagagggcccatctaatcgagatgaaaacatgtgaatg
gccaaagtcccacacattgtgggcagatggaatagaagagagtgatctgatcattcccaagtctttagctgggccactcagccatcacaataccaga
gagggctacaggacccaaatgaaagggccatggcacagtgaagagcttgaaattcggtttgaggaatgcccgggcactaaggtccacgtggag
gaaacatgtggaacaagaggaccatctctgagatcaaccactgcaagcggaagggtgatcgaggaatggtgctgcagggagtgcacaatgccc
ccactgtcgttccgggctaaagatggctgttggtatggaatggagataaggcccaggaaagaaccagaaagcaacttagtaaggtcagtggtgact
gcaggatcaactgatcacatggatcacttctcccttggagtgcttgtgattctgctcatggtgcaggaagggctgaagaagagaatgaccacaaaga
tcatcataagcacatcaatggcagtgctggtagctatgatcctgggaggattttcaatgagtgacctggctaagcttgcaattttgatgggcgccacct
tcgcggaaatgaacactggaggagatgtagctcatctggcgctgatagcggcattcaaagtcagaccagcgttgctggtatctttcatcttcagagct
aattggacaccccgtgaaagcatgctgctggccttggcctcgtgtcttttgcaaactgcgatctccgccttggaaggcgacctgatggttctcatcaat
ggttttgctttggcctggttggcaatacgagcgatggttgttccacgcactgataacatcaccttggcaatcctggctgctctgacaccactggcccgg
ggcacactgcttgtggcgtggagagcaggccttgctacttgcgggggggtttatgctcctctctctgaagggaaaaggcagtgtgaagaagaactta
ccatttgtcatggccctgggactaaccgctgtgaggctggtcgaccccatcaacgtggtgggactgctgttgctcacaaggagtgggaagcggag
ctggcccccctagcgaagtactcacagctgttggcctgatatgcgcattggctggagggttcgccaaggcagatatagagatggctgggcccatgg
ccgcggtcggtctgctaattgtcagttacgtggtctcaggaaagagtgtggacatgtacattgaaagagcaggtgacatcacatgggaaaaagatg
cggaagtcactggaaacagtccccggctcgatgtggcgctagatgagagtggtgatttctccctggtggaggatgacggtccccccatgagagag
atcatactcaaggtggtcctgatgaccatctgtggcatgaacccaatagccataccctttgcagctggagcgtggtacgtatacgtgaagactggaa
aaaggagtggtgctctatgggatgtgcctgctcccaaggaagtaaaaaagggggagaccacagatggagtgtacagagtaatgactcgtagactg
ctaggttcaacacaagttggagtgggagttatgcaagagggggtctttcacactatgtggcacgtcacaaaaggatccgcgctgagaagcggtga
agggagacttgatccatactggggagatgtcaagcaggatctggtgtcatactgtggtccatggaagctagatgccgcctgggacgggcacagcg
aggtgcagctcttggccgtgccccccggagagagagcgaggaacatccagactctgcccggaatatttaagacaaaggatggggacattggagc
ggttgcgctggattacccagcaggaacttcaggatctccaatcctagacaagtgtgggagagtgataggactttatggcaatggggtcgtgatcaaa
aatgggagttatgttagtgccatcacccaagggaggagggaggaagagactcctgttgagtgcttcgagccttcgatgctgaagaagaagcagct
aactgtcttagacttgcatcctggagctgggaaaaccaggagagttcttcctgaaatagtccgtgaagccataaaaacaagactccgtactgtgatctt
agctccaaccagggttgtcgctgctgaaatggaggaagcccttagagggcttccagtgcgttatatgacaacagcagtcaatgtcacccactctgga
acagaaatcgtcgacttaatgtgccatgccaccttcacttcacgtctactacagccaatcagagtccccaactataatctgtatattatggatgaggccc
acttcacagatccctcaagtatagcagcaagaggatacatttcaacaagggttgagatgggcgaggcggctgccatcttcatgaccgccacgccac
caggaacccgtgacgcatttccggactccaactcaccaattatggacaccgaagtggaagtcccagagagagcctggagctcaggctttgattgg
gtgacggatcattctggaaaaacagtttggtttgttccaagcgtgaggaacggcaatgagatcgcagcttgtctgacaaaggctggaaaacgggtc
atacagctcagcagaaagacttttgagacagagttccagaaaacaaaacatcaagagtgggactttgtcgtgacaactgacatttcagagatgggc
gccaactttaaagctgaccgtgtcatagattccaggagatgcctaaagccggtcatacttgatggcgagagagtcattttggctggacccatgcctgt
cacacatgccagcgctgcccagaggagggggcgcataggcaggaatcccaacaaacctggagatgagtatctgtatggaggtgggtgcgcaga
gactgacgaagaccatgcacactggcttgaagcaagaatgctccttgacaatatttacctccaagatggcctcatagcctcgctctatcgacctgagg
ccgacaaagtagcagccattgagggagagttcaagcttaggacggagcaaaggaagacctttgtggaactcatgaaaagaggagatcttcctgttt
ggctggcctatcaggttgcatctgccggaataacctacacagatagaagatggtgctttgatggcacgaccaacaacaccataatggaagacagtg
tgccggcagaggtgtggaccagacacggagagaaaagagtgctcaaaccgaggtggatggacgccagagtttgttcagatcatgcggccctga
agtcattcaaggagtttgccgctgggaaaagaggagcggcttttggagtgatggaagccctgggaacactgccaggacacatgacagagagattc
caggaagccattgacaacctcgctgtgctcatgcgggcagagactggaagcaggccttacaaagccgcggcggcccaattgccggagaccctа
gagaccattatgcttttggggttgctgggaacagtctcgctgggaatctttttcgtcttgatgaggaacaagggcatagggaagatgggctttggaat
ggtgactcttggggccagcgcatggctcatgtggctctcggaaattgagccagccagaattgcatgtgtcctcattgttgtgttcctattgctggtggt
gctcatacctgagccagaaaagcaaagatctccccaggacaaccaaatggcaatcatcatcatggtagcagtaggtcttctgggcttgattaccgcc
aatgaactcggatggttggagagaacaaagagtgacctaagccatctaatgggaaggagagaggagggagcaaccataggattctcaatggaca
ttgacctgcggccagcctcagcttgggccatctatgctgccttgacaactttcattaccccagccgtccaacatgcagtgaccacttcatacaacaact
actccttaatggcgatggccacgcaagctggagtgttgtttggtatgggcaaagggatgccattctacgcatgggactttggagtcccgctgctaatg
ataggttgctactcacaattaacacccctgaccctaatagtggccatcattttgctcgtggcgcactacatgtacttgatcccagggctgcaggcagca
gctgcgcgtgctgcccagaagagaacggcagctggcatcatgaagaaccctgttgtggatggaatagtggtgactgacattgacacaatgacaatt

gacccccaagtggagaaaaagatgggacaggtgctactcatagcagtagcagtctccagcgccatactgtcgcggaccgcctgggggtggggg
gaggctggggccctgatcacagccgcaacttccactttgtgggaaggctctccgaacaagtactggaactcctctacagccacttcactgtgtaaca
tttttaggggaagttacttggctggagcttctctaatctacatagtaacaagaaacgctggcttggtcaagagacgtgggggtggaacaggagagac
cctgggagagaaatggaaggcccgcttgaaccagatgtcggccctggagttctactcctacaaaaagtcaggcatcaccgaggtgtgcagagaa
gaggcccgccgcgccctcaaggatggtgtggcaacgggaggccatgctgtgtcccgaggaagtgcaaagctgagatggttggtggagcgggg
atacctgcagccctatggaaaggtcattgatcttggatgtggcagagggggctggagttactacgccgccaccatccgcaaagttcaagaagtgaa
aggatacacaaaaggaggccctggtcatgaagaacccgtgttggtgcaaagctatgggtggaacatagtccgtcttaagagtggggtggacgtctt
tcatatggcggctgagccgtgtgacacgttgctgtgtgacataggtgagtcatcatctagtcctgaagtggaagaagcacggacgctcagagtcctc
tccatggtgggggattggcttgaaaaaagaccaggagccttttgtataaaagtgttgtgcccatacaccagcactatgatggaaaccctggagcgac
tgcagcgtaggtatgggggaggactggtcagagtgccactctcccgcaactctacacatgagatgtactgggtctctggagcgaaaagcaacacc
ataaaaagtgtgtccaccacgagccagctcctcttggggcgcatggacgggcctaggaggccagtgaaatatgaggaggatgtgaatctcggctc
tggcacgcgggctgtggtaagctgcgctgaagctcccaacatgaagatcattggtaaccgcattgaaaggatccgcagtgagcacgcggaaacg
tggttctttgacgagaaccacccatataggacatgggcttaccatggaagctatgaggcccccacacaagggtcagcgtcctctctaataaacggg
gttgtcaggctcctgtcaaaaccctgggatgtggtgactggagtcacaggaatagccatgaccgacaccacaccgtatggtcagcaaagagttttca
aggaaaaagtggacactagggtgccagacccccaagaaggcactcgtcaggttatgagcatggtctcttcctggttgtggaaagagctaggcaaa
cacaaacggccacgagtctgtaccaaagaagagttcatcaacaaggttcgtagcaatgcagcattaggggcaatatttgaagaggaaaaagagtg
gaagactgcagtggaagctgtgaacgatccaaggttctgggctctagtggacaaggaaagagagcaccacctgagaggagagtgccagagttgt
gtgtacaacatgatgggaaaaagagaaaagaaacaaggggaatttggaaaggccaagggcagccgcgccatctggtatatgtggctaggggct
agatttctagagttcgaggcccttggattcttgaacgaggatcactggatggggagagagaactcaggaggtggtgttgaagggctgggattacaa
agactcggatatgtcctagaagagatgagtcgcataccaggaggaaggatgtatgcagatgacactgctggctgggacacccgcatcagcaggtt
cgatctggagaatgaagctctaatcaccaaccaaatggagaaagggcatagggccttggcattggccataatcaagtacacataccaaaacaaagt
ggtaaaggtccttagaccagctgaaaaagggaaaacagttatggacattatttcgagacaagaccaaagggggagcggacaagttgtcacttacg
ctcttaacacatttaccaacctagtggtgcaactcattcggaatatggaggctgaggaagttctagagatgcaagacttgtggctgctgcggaggtca
gagaaagtgaccaactggttgcagagcaacggatgggataggctcaaacgaatggcagtcagtggagatgattgcgttgtgaagccaattgatga
taggtttgcacatgccctcaggttcttgaatgatatgggaaaagttaggaaggacacacaagagtggaaaccctcaactggatgggacaactggga
agaagttccgttttgctcccaccacttcaacaagctccatctcaaggacgggaggtccattgtggttccctgccgccaccaagatgaactgattggcc
gggcccgcgtctctccaggggcgggatggagcatccgggagactgcttgcctagcaaaatcatatgcgcaaatgtggcagctcctttatttccaca
gaagggacctccgactgatggccaatgccatttgttcatctgtgccagttgactgggttccaactgggagaactacctggtcaatccatggaaaggg
agaatggatgaccactgaagacatgcttgtggtgtggaacagagtgtggattgaggagaacgaccacatggaagacaagaccccagttacgaaat
ggacagacattccctatttgggaaaaagggaagacttgtggtgtggatctctcatagggcacagaccgcgcaccacctgggctgagaacattaaaa
acacagtcaacatggtgcgcaggatcataggtgatgaagaaaagtacatggactacctatccacccaagttcgctacttgggtgaagaagggtcta
cacctggagtgctgtaagtttaaaccctcccccccctaacgttactggccgaagccgcttggaataaggccggtgtgcgtttgtctatatgttattttcc
accatattgccgtcttttggcaatgtgagggcccggaaacctggccctgtcttcttgacgagcattcctaggggtctttcccctctcgccaaaggaatg
caaggtctgttgaatgtcgtgaaggaagcagttcctctggaagcttcttgaagacaaacaacgtctgtagcgaccctttgcaggcagcggaaccccc
cacctggcgacaggtgcctctgcggccaaaagccacgtgtataagatacacctgcaaaggcggcacaaccccagtgccacgttgtgagttggata
gttgtggaaagagtcaaatggctctcctcaagcgtattcaacaaggggctgaaggatgcccagaaggtaccccattgtatgggatctgatctgggg
cctcggtgcacatgctttacatgtgtttagtcgaggttaaaaaaacgtctaggccccccgaaccacggggacgtggttttcctttgaaaaacacgatg
ataatatggtcttcacactcgaagatttcgttggggactggcgacagacagccggctacaacctggaccaagtccttgaacagggaggtgtgtcca
gtttgtttcagaatctcggggtgtccgtaactccgatccaaaggattgtcctgagcggtgaaaatgggctgaagatcgacatccatgtcatcatcccgt
atgaaggtctgagcggcgaccaaatgggccagatcgaaaaaatttttaaggtggtgtaccctgtggatgatcatcactttaaggtgatcctgcactat
ggcacactggtaatcgacggggttacgccgaacatgatcgactatttcggacggccgtatgaaggcatcgccgtgttcgacggcaaaaagatcac
tgtaacagggaccctgtggaacggcaacaaaattatcgacgagcgcctgatcaaccccgacggctccctgctgttccgagtaaccatcaacggag
tgaccggctggcggctgtgcgaacgcattctggcgggtagcatgcagatcttcgtgaagaccctgacgggcaagaccatcactcttgaggtcgag
cccagtgacaccatcgagaatgtcaaggccaagatccaagacaaggaaggcatcccacctgaccagcagaggctgatattcgcgggcaaacag
ctggaggatggccgcaccctgtccgactacaacatccagaaagagtccaccttgcacctggtgctgcgtctccgcggtggtatgattgaacaagat
ggattgcacgcaggttctccggccgcttgggtggagaggctattcggctatgactgggcacaacagacaatcggctgctctgatgccgccgtgttc
cggctgtcagcgcaggggcgcccggttctttttgtcaagaccgacctgtccggtgccctgaatgaactgcaggacgaggcagcgcggctatcgtg
gctggccacgacgggcgttccttgcgcagctgtgctcgacgttgtcactgaagcgggaagggactggctgctattgggcgaagtgccggggcag
gatctcctgtcatctcaccttgctcctgccgagaaagtatccatcatggctgatgcaatgcggcggctgcatacgcttgatccggctacctgcccattc
gaccaccaagcgaaacatcgcatcgagcgagcacgtactcggatggaagccggtcttgtcgatcaggatgatctggacgaagagcatcagggg
ctcgcgccagccgaactgttcgccaggctcaaggcgcggatgcccgacggcgaggatctcgtcgtgacccacggcgatgcctgcttgccgaata
tcatggtggaaaatggccgcttttctggattcatcgactgtggccggctgggtgtggcggaccgctatcaggacatagcgttggctacccgtgatatt
gctgaagagcttggcggcgaatgggctgaccgcttcctcgtgctttacggtatcgccgctcccgattcgcagcgcatcgccttctatcgccttcttga
cgagttcttctgagaattcgcaccaatcttaatgttgtcaggcctgctagtcagccacagcttggggaaagctgtgcagcctgtgacccccccagga
gaagctgggaaaccaagcctatagtcaggccgagaacgccatggcacggaagaagccatgctgcctgtgagcccctcagaggacactgagtca

aaaaaccccatgcgcttggaggcgcaggatgggaaaagaaggtggcgaccttccccacccttcaatctggggcctgaactggagatcagctgtg
gatctccagaagagggactagtggttagaggagaccccccggaaaacgcaaaacagcatattgacgctgggaaagaccagagactccatgagtt
tccaccacgctggccgccaggcacagatcgccgaatagcggcggccggtgtggggaaatccatgggtctt

b

Feature	Location (nuc range)	Size (nuc)
ZIKV 5'	1 - 107	107
Nucleocapsid	108 - 200	93
envelope	201 - 296	96
NS1-NS5	297 - 8,186	7,890
EMCV-IRES	8,187 - 8,759	573
NanoLuc	8,760 - 9,272	513
Ubiquitine region	9,279 - 9,506	228
Neomycine resistance gene	9,507 - 10,301	795
ZIKV 3'	10,308 - 10,736	429

Supplementary Figure 4 | ZIKV replicon nucleotide sequence and annotated features. a Full nucleotide sequence of the engineered ZIKV replicon used in this study. **b** Annotated features of the replicon sequence, including untranslated regions (UTRs), structural and non-structural elements and reporter gene insertion sites.

a NS2B alignment

```
ZKV           SWPPSEVLTAVGLICALAGGFAKADIE-MAGPMAAVGLLIVSYVVSGKSVDMYIERAGDI 59
DENV2         SWPLNEAIMAVGMVSILASSLLKNDIP-MTGPLVAGGLLTVCYVLTGRSADLELERAADV 59
WNV           GWPATEVMTAVGLMFAIVGGLAELDIDSMAIPMTIAGLMFAAFVISGKSTDMWIERTADI 60
JEV           -----------------------------------------------------LERAADI 7
YFV           SIPVNEALAAAGLVG-VLAGLAFQDMENFLGPIAVGGILMMLVSVAGRVDGLELRKLGEI 59

ZKV           TWEKDAEVTGNSPRLDVALDESGDFSLVEDDGPPMREIILKVVLMTICGMNPIAIP-FAA 118
DENV2         KWEDQAEISGSSPILSITISEDGSMSIKNEEEEQTLTILIRTGLLVISGLFPVSIP-ITA 118
WNV           SWESDAEITGSSERVDVRLDDDGNFQLMNDPGAPWKIWMLRMVCLAISAYTPWAILPSVV 120
JEV           SWEMDAAITGSSRRLDVKLDDDGDFHLIDDPGV--------------------------- 40
YFV           SWEEEAEISGSSSRYDVALSEQGEFKLLSEDKVPWDQIVMTSLALVGAAIHPFALL-LVL 118

ZKV           GAWYVYVKTGKR-          130
DENV2         AAWYLWEVKKQR-          130
WNV           GFWIT-LQYTKRG          132
JEV           -------------          40
YFV           GGWILHIKGARR-          130
```

b NS3 alignment

```
                                                                  *
ZKV           -SGALWDVPAPKEVKKG-ETTDGVYRVMTRRLLGSTQVGVGVMQEGVFHTMWHVTKGSAL 58
DENV2         -AGVLWDVPSPPPVGKA-ELEDGAYRIKQKGILGYSQIGAGVYKEGTFHTMWHVTRGAVL 58
WNV           -GGVLWDTPSPKEYKKG-DTTTGVYRIMTRGLLGSYQAGAGVMVEGVFHTLWHTTKGAAL 58
JEV           -GGVFWDTPSPKPCSKG-DTTTGVYRIMARGILGTYQAGVGVMYENVFHTLWHTTRGAAI 58
YFV           SGDVLWDIPTPKVIEECEHLEDGIYGIFQSTFLGASQRGVGVAQGGVFHTMWHVTRGAFL 60
                              *             ǂ     ǂ                 ǂ
ZKV           RSGEGRLDPYWGDVKQDLVSYCGPWKLDAAWDGHSEVQLLAVPPGERARNIQTLPGIFKT 118
DENV2         MHKGKRIEPSWADVKKDLISYGGGWKLEGEWKEGEEVQVLALEPGKNPRAVQTKPGLFKT 118
WNV           MSGEGRLDPYWGSVKEDRLCYGGPWKLQHKWNGQDEVQMIVVEPGKNVKNVQTKPGVFKT 118
JEV           MSGEGKLTPYWGSVKEDRIAYGGPWRFDRKWNGTDDVQVIVVEPGKAAVNIQTKPGVFRT 118
YFV           LRNGKKLVPSWASVKEDLVAYGGSWKLDGRWDGEEEVQLIAAVPGKSVVNVQTKPSLFKV 120
                      §        *   ǂ       ǂ § §   156
ZKV           K-DGDIGAVALDYPAGTSGSPILDKCGRVIGLYGNGVVIKNGSYVSAITQGRREEETPVE 177
DENV2         N-AGTIGAVSLDFSPGTSGSPIIDKKGKVVGLYGNGVVTRSGAYVSAIAQTEKSIEDNPE 177
WNV           P-EGEIGAVTLDFPTGTSGSPIVDKNGDVIGLYGNGVIMPNGSYISAIVQGERMDEPIPA 177
JEV           P-FGEVGAVSLDYPRGTSGSPILDSNGDIIGLYGNGVELGDGSYVSAIVQGDRQEEPVPE 177
YFV           KNGGEIGAVALDYPSGTSGSPIVNRNGEVVGLYGNGILVGDNSFVSAISQTELKEESKEE 180

ZKV           C           178
DENV2         I           178
WNV           G           178
JEV           A           178
YFV           L           181
```

Supplementary Figure 5 | Multiple sequence alignment of orthoflavivirus NS2B and NS3 proteins. **a** Sequence alignment of NS2B protease cofactors from ZIKV, DENV2, WNV, JEV, and YFV. **b** Sequence alignment of NS3 proteases from the same viruses. Catalytic triad residues (H51, D75, and S135) are highlighted in bold and purple and marked with an asterisk (*). Residue 156, associated with resistance in mutational studies, is boxed. Residues involved in binding to *R*-(+)-IRBM-Z-1, as defined by the ZIKV NS2B-NS3 crystal structure, are marked with ǂ (for residues contributing to the hydrophobic pocket) and § (for residues forming hydrogen bonds).

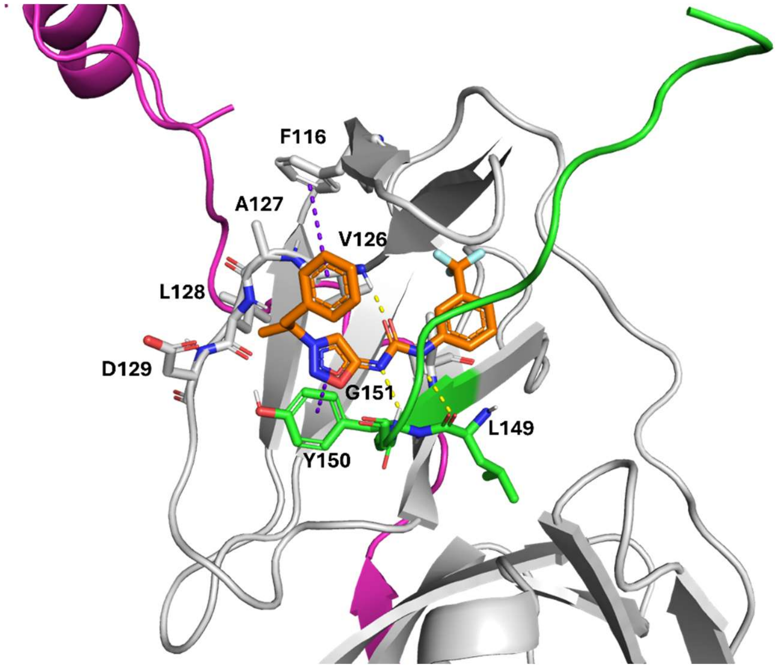

Supplementary Figure 6 | Docking pose of *S*-(-)-IRBM-Z-1 enantiomer in complex with the ZIKV NS2B-NS3 protease. The *S*-enantiomer of IRBM-Z-1 (orange and blue sticks) maintains key interactions with the protease (NS3 in white, NS2B in pink and the NS3 C-terminal segment in green), with an additional π-stacking interaction with F116, not observed in the *R*-(+)-IRBM-Z-1 co-crystal structure due to the presence of a second ligand molecule.

	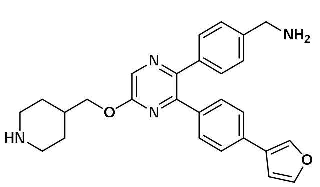 **Compound 9**[6]	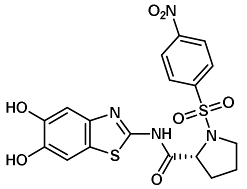 **Compound 12R**[7]	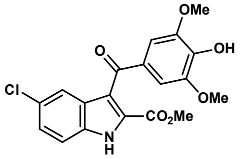 **Compound 1**[8]
**Biological Data Literature** IC_{50} (μM)	Linked-ZVpro 0.2±0.01<br>ZIKV-FLR EC_{68} 0.30-0.60<br>DV2pro 0.59±0.02<br>DV3pro 0.52±0.06<br>WVpro 0.78±0.02	ZIKVpro 0.32±0.05<br>DV2pro 10.0±1.0	ZIKVpro 158±25<br>ZIKV-cell-infection<br>EC_{50} 13.9±1.1
**Biological Data IRBM** IC_{50} (μM)	ZIKVpro 0.32±0.05<br>ZIKVpro 10.6±1.1<br>ZIKV-repl EC_{50}14.9±1.7<br>DV2pro 13.7±8.4	ZIKVpro 0.9±0.4<br>ZIKV-repl EC_{50}>16<br>DV2pro 13.7±8.4	Not determined
**MoA Studies**	X-Ray DENV2 NS2B-NS3<br>PDB ID: 6OM0<br>Allosteric pocket	Predicted binding mode.<br>NS2B-NS3 Allosteric pocket	Predicted binding mode.<br>NS2B-NS3 Allosteric pocket
***In vivo* ADME**	ZIKV C57BL/6. IP, QD, 9 days, 15 mpk.<br>Plasma and brain RNA log reduction: 1.7 and 1.9	Not reported	ZIKV ICR. IP, QD, 5 days, 1 mpk. Brain RNA log reduction: 1.5

Supplementary Table 2 | Comparison of biological and ADME data of known ZIKV NS2B-NS3 allosteric inhibitors. Replicon assays were carried out using Vero cells. Data of the biochemical assays are expressed as IC_{50} values at mM concentration. IC_{50}, 50% inhibition concentration. Data of the cell-based assays are expressed as EC_{50} values at μM concentrations. EC_{50}, 50% effective concentration. All data represent the mean ± SD from at least six independent experiments.

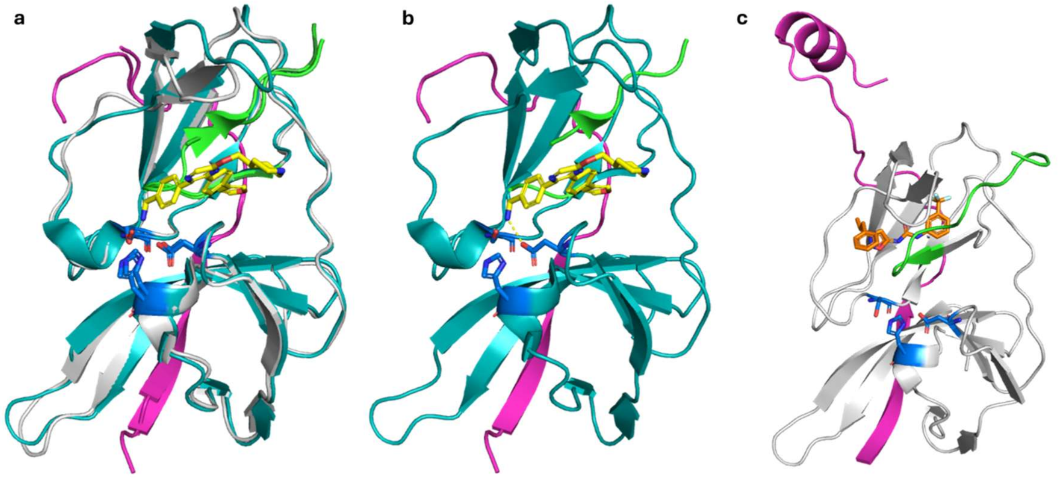

Supplementary Figure 7 | Comparison of *R*-(+)-IRBM-Z-1 and compound 9 binding sites. The ZIKV protease inhibitor compound 9 from Yao et al.[6], co-crystallized in complex with the DENV2 protease (PDB ID: 6MO0), and *R*-(+)-IRBM-Z-1 occupy distinct, yet partially overlapping, allosteric sites through the C-terminal segment of NS3. **a** Superposition of the DENV2 NS2B-NS3/compound 9 complex (blue cartoons, NS2B in pink, NS3 C-terminal segment in light green, compound 9 in yellow; PDB ID: 6MO0) with the crystal structure of the apo ZIKV NS2B-NS3 protease in an open conformation (white cartoon, NS2B in pink, NS3 C-terminal segment in dark green; PDB ID: 5GXJ). The NS3 C-terminal regions align closely (RMSD = 0.325 Å). Side-to-side comparison of the X-ray structure of DENV2 NS2B-NS3/compound 9 complex (blue cartoons, NS2B in pink, NS3 C-terminal segment in light green, compound 9 in yellow; PDB ID: 6MO0). **b** Compound 9 forms a hydrogen bond with D75, a member of the catalytic triad, an interaction not observed with IRBM-Z-2. The DENV2 NS3 C-terminal region is partially unresolved (residues 152–164). Catalytic residues are shown as dark blue sticks. **c** The ZIKV NS2B-NS3 structure (white cartoon, NS2B in pink, NS3 C-terminal segment in green) in complex with *R*-(+)-IRBM-Z-1 (orange sticks).

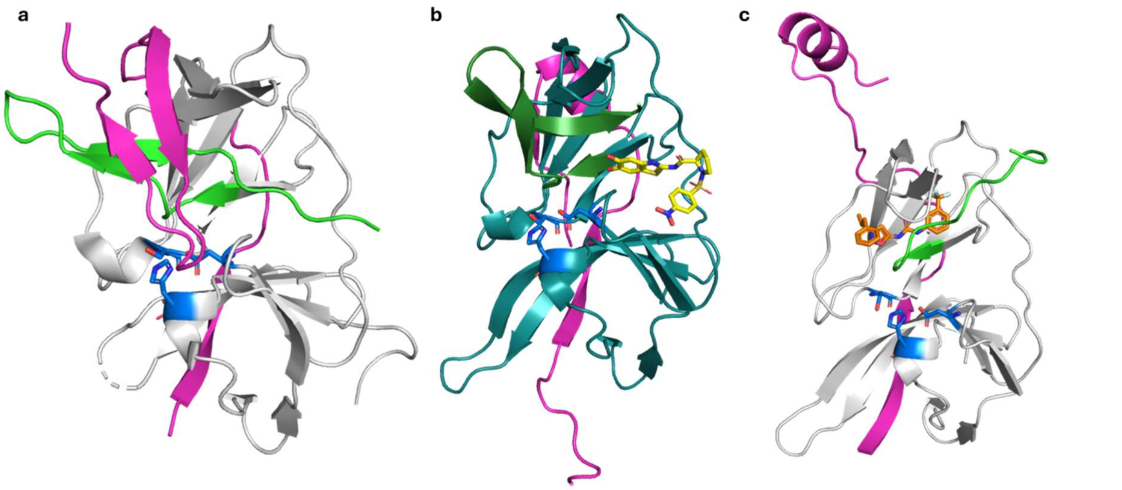

Supplementary Figure 8 | Comparison of *R*-(+)-IRBM-Z-1 and compound 12R binding sites. The binding site of compound 12R from Milles et al.[7] identified via docking on the DENV2 NS2B-NS3 protease (PDB ID: 2FOM[9]) is different compared to *R*-(+)-IRBM-Z-1 and IRBM-Z-2 (Figure 2e, 3c). In particular, the conformation of DENV2 NS2B-NS3 is compatible with a closed conformation of the ZIKV NS2B-NS3 protease, as the NS3 C-terminal segment is stabilized in a β-sheet conformation. **a** Apo structure of ZIKV NS2B-NS3 in a closed conformation (PDB ID: 5GPI, white cartoons, NS2B in pink, NS3 C-terminal segment in green). Residue I156 forms hydrogen bonds (yellow dashes) with G159 and S160 (residues involved in the hydrogen bond are shown as sticks). **b** Docking pose of compound 12R (yellow sticks) in complex with the DENV2 NS2B-NS3 protease (PDB ID: 2FOM[39], blue cartoons, NS2B in pink, NS3 C-terminal segment in dark green). Residue T156 forms an extended hydrogen bond network compared to I156 in ZKV NS3. **c** X-ray structure of ZIKV NS2B-NS3 (NS2B in pink, NS3 C-terminal segment in green) in complex with *R*-(+)-IRBM-Z-2 (orange sticks).

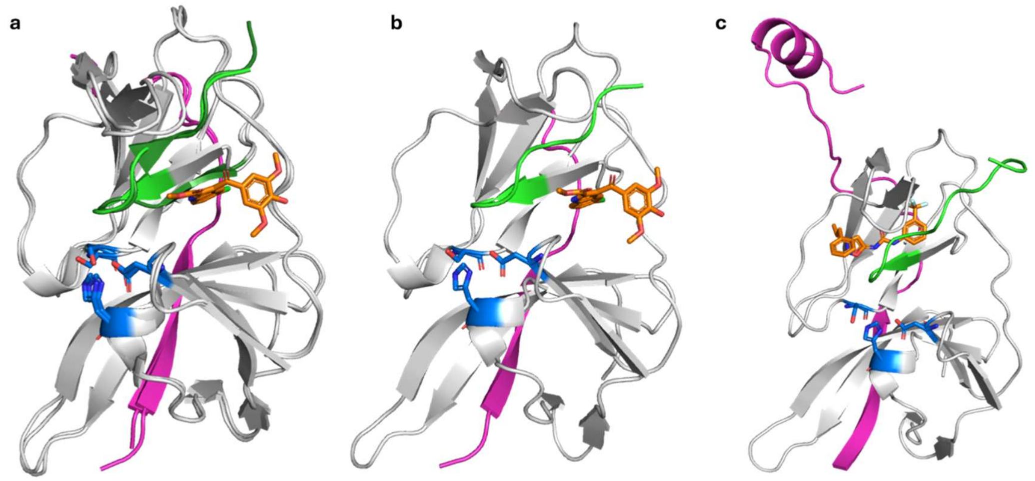

Supplementary Figure 9 | Comparison of *R*-(+)-IRBM-Z-1 and compound 1 binding sites. Compound 1, from Coluccia et al.[8], identified via docking on the ZIKV NS2B-NS3 protease (PDB ID: 5TLV), occupies a different allosteric site on ZIKV NS2B-NS3 compared to *R*-(+)-IRBM-Z-1 and IRBM-Z-2. The structure used for the docking studies is in an open conformation similar to 5GXJ, in which the NS3 C-terminal segment partially occupies the binding site of *R*-(+)-IRBM-Z-1. **a** Superposition of the docking pose of compound 1 (orange sticks) using the 5TLV X-ray structure (white cartoons, NS2B in pink, NS3 C-terminal segment in light green), with the ZIKV NS2B-NS3 apo structure in an open conformation (PDB ID: 5GXJ, transparent cartoons, NS2B in pink, NS3 C-terminal segment in dark green). Side-to-side comparison of the reproduced docking pose of compound 1 (orange sticks) in complex with ZIKV NS2B-NS3 (**b**) and the X-ray structure of ZIKV NS2B-NS3 in complex with *R*-(+)-IRBM-Z-1 (orange sticks) (**c**).

	9TPG (R-(+)-IRBM-Z-1)[1]	9IBY (IRBM-Z-2)[1]
**Data collection**		
Space group	I 2 2 2	P 1 2 1
Cell dimensions		
a, b, c (Å)	59.58; 91.55; 103.84	61.79; 57.47; 72.45
α, β, γ (°)	90.0; 90.0; 90.0	90.0; 96.4; 90.0
Resolution (Å)	2.48 (2.69-2.48)*	2.29 (2.58-2.29)*
R_{sym} or R_{merge}	0.13	0.14
$I / \sigma I$	9.1 (1.3)	6.8 (1.3)
Completeness (%)[2]	90.9 (58.7)[3]	89.6 (53.9)[4]
Redundancy	7.7 (7.8)	6.6 (6.6)
**Refinement**		
Resolution (Å)	68.67-2.48	44.96-2.29
No. reflections	5460 / 269	13630 / 733
R_{work} / R_{free}	24.7/29.6	18.9/23.6
No. atoms		
Protein	1369	2474
Ligand/ion	56/10	68/12
Water	31	54
B-factors		
Protein	59.6	54.3
Ligand/ion	65.9/96.5	45.0/64.3
Water	46.9	44.3
R.m.s. deviations		
Bond lengths (Å)	0.003	0.004
Bond angles (°)	1.07	1.28

Supplementary Table 3 | Data collection and refinement statistics (anisotropic statistics, molecular replacement)[1] for the ZIKV protease and *R*-(+)-IRBM-Z-1 complex (PDB ID 9TPG) and the for the ZIKV protease and IRBM-Z-2 complex (PDB ID 9IBY). [1] Values in parentheses are for highest-resolution shell. Data for 9TPG *R*-(+)-IRBM-Z-1 were collected at SWISS LIGHT SOURCE (SLS, Villigen, Switzerland) and for 9IBY IRBM-Z-2 were collected at European Synchrotron Radiation Facility (ESRF, Grenoble, France). [2] Completeness is calculated after anisotropic truncation. [3] Isotropic data extended to 3.01 Å with 100 % completeness; anisotropic truncation applied prior to refinement. [4] Isotropic data extended to 2.67 Å with 98.6% completeness; anisotropic truncation applied prior to refinement.

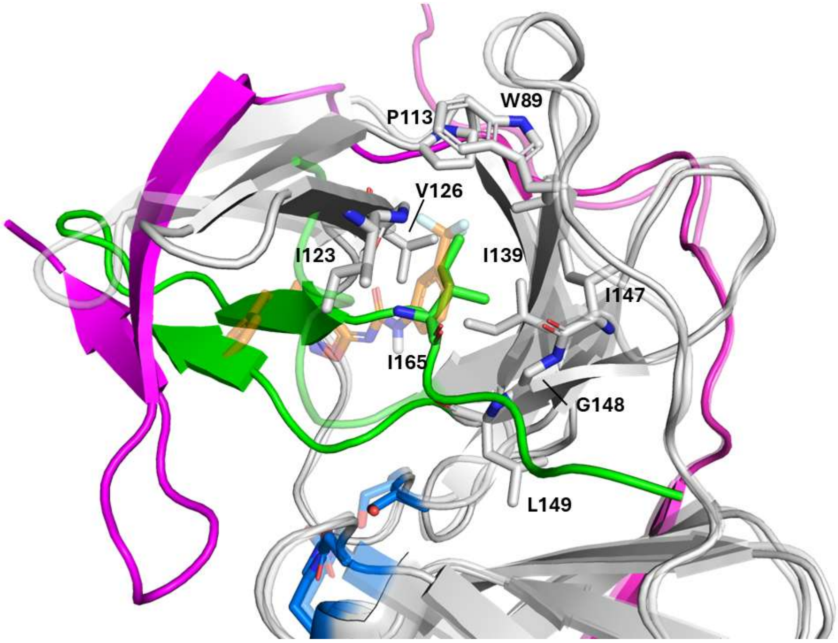

Supplementary Figure 10 | Superposition of the ZIKV NS2B-NS3 - *R*-(+)-IRBM-Z-1 complex with the apo ZIKV NS2B-NS3 closed conformation. Overlay of the *R*-(+)-IRBM-Z-1-bound structure (transparent cartoons; IRBM-Z-1 in orange sticks) and the apo closed conformation of ZIKV NS2B–NS3 (opaque cartoons; PDB ID: 5GPI). NS2B is shown in pink, and the NS3 N-terminal segment in green.

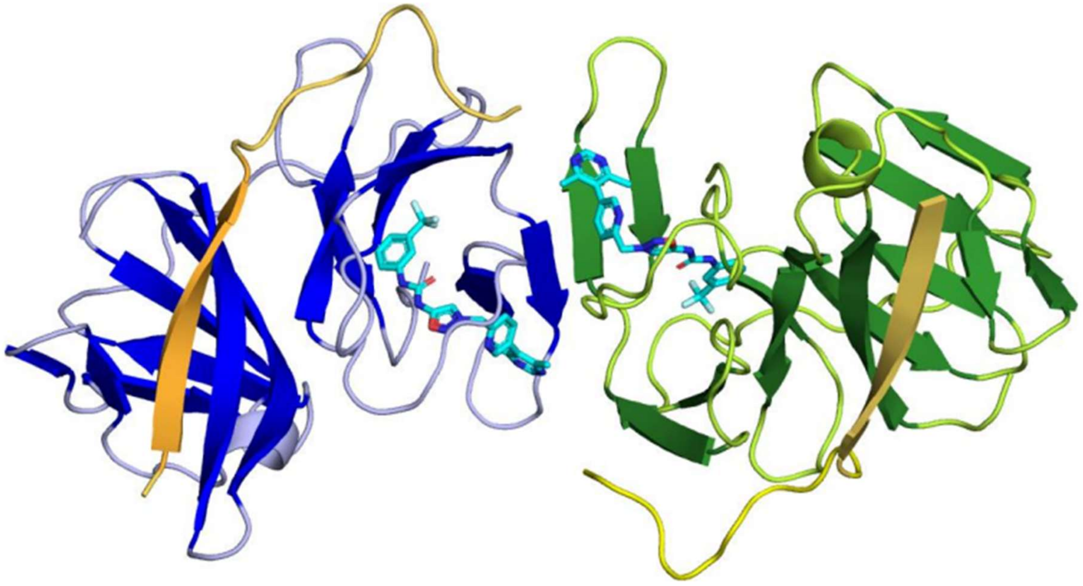

Supplementary Figure 11 | Crystal structure of ZIKV NS2B–NS3 in complex with IRBM-Z-2. Ribbon representation of the ZIKV NS2B–NS3 dimer (PDB ID: 9IBY), with NS2B in orange and NS3 in blue and green. IRBM-Z-2 is shown as a stick model (cyan).

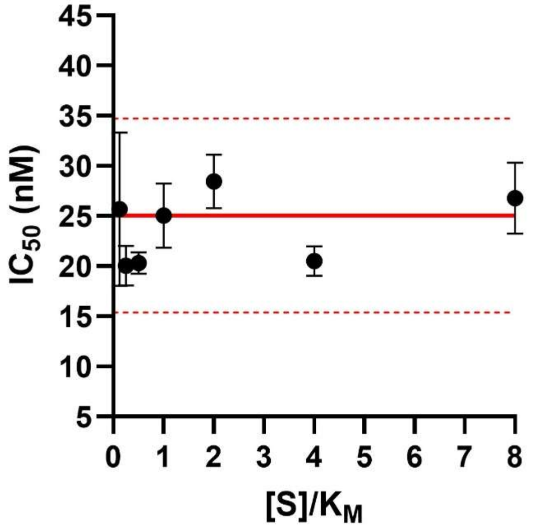

$[S]/K_M$	IRBM-Z-1 IC_{50}(nM)	IRBM-Z-2 IC_{50}(nM)
8	1006 ± 83	26.8 ± 3.5
4	941 ± 41	20.5 ± 1.5
2	1256 ± 172	28.4 ± 2.7
1	1122 ± 137	25.1 ± 3.2
0.5	1227 ± 177	20.3 ± 1.1
0.25	1355 ± 148	20.1 ± 1.9
0.125	1484 ± 228	25.7 ± 7.6

Supplementary Figure 12 | Biophysical characterization of IRBM-Z-1 and IRBM-Z-2. Graphic of mechanism of action for **IRBM-Z-2** on the NS2B-NS3 protease. The solid red line represents the IC_{50} value at substrate concentration $[S] = K_M$, dashed red lines indicate the confidence interval (IC_{50} $[S] = K_M \pm 3$ SD). Table represents the IRBM-Z-1 and IRBM-Z-2 IC_{50} values at increasing $[S]/K_M$ ratios. The IC_{50} values are reported as the mean and standard deviation of six different replicates.

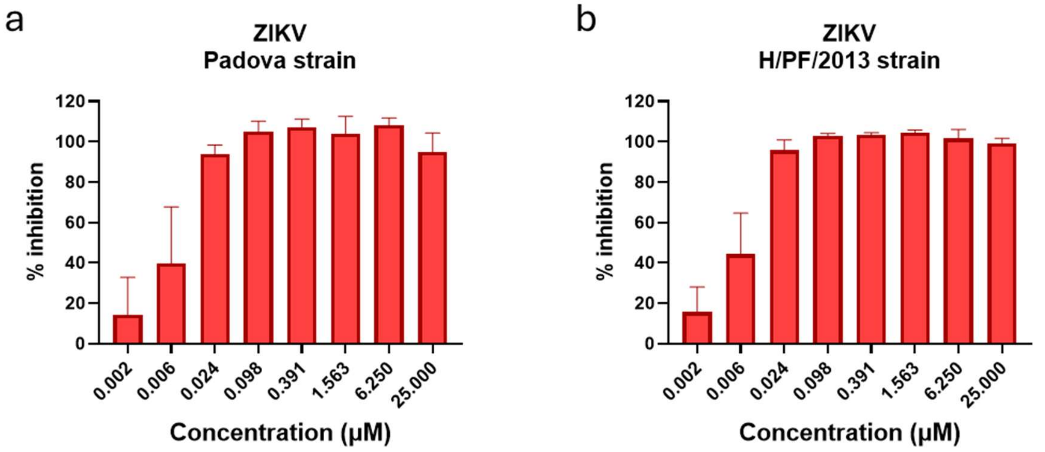

Supplementary Figure 13 | ZIKV infection assay of IRBM-Z-2 in HuH-7 cells. Dose–response curves showing inhibition of ZIKV infection by IRBM-Z-2 in HuH-7 cells infected with the Padova (a) and H/PF/2013 (b) strains. Inhibition values are expressed as percent reduction in infected cells, normalized to DAPI-positive cell counts (set to 4000 per well). Mock-infected controls were used to establish background levels of immunostaining. Cytotoxicity was evaluated by MTT assay. For both ZIKV strains, the estimated IC_{50} ranged from 6 to 20 nM.

Hepatocyte stability						
compound	Clint (µl/min/10^6 cells)			$T_{1/2}$ (h)		
	Human	Rat	Dog	Human	Rat	Dog
IRBM-Z-2	3.7	10.3	2.4	60	17	34

Supplementary Table 4 | Hepatocyte stability of IRBM-Z-2. All data from biological assays are the results of at least two independent experiments.

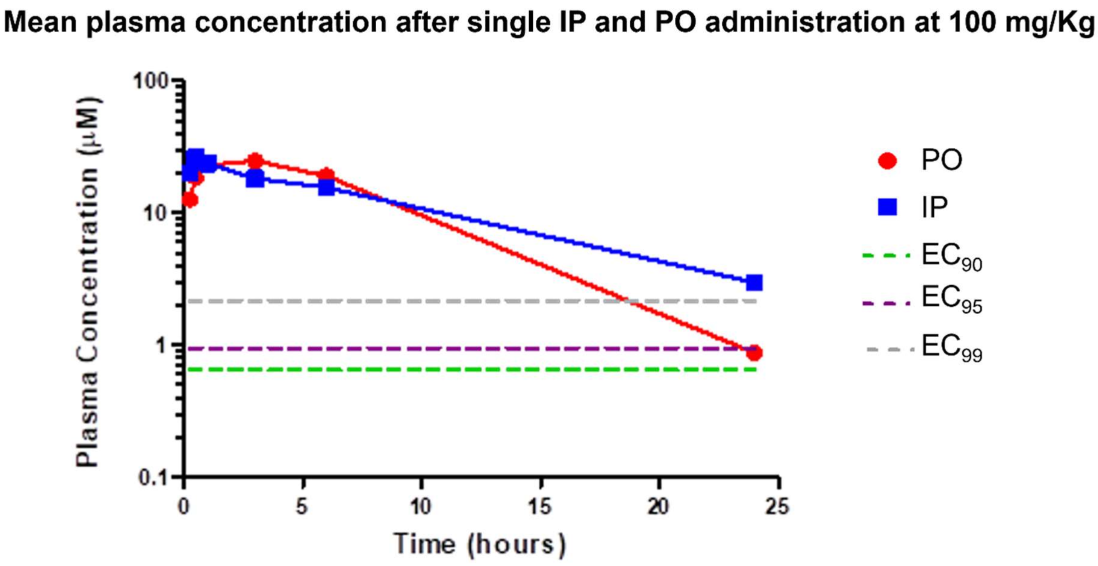

**Route**	**Dose** (mpk)	**Matrix**	**C$_{max}$** (μM/mL)	**T$_{max}$** (h)	**AUC** (μM*Hours)
IP	100 QD	Plasma	28	0.7	314
PO			27	3.3	315

Supplementary Figure 14 | *In vivo* pharmacokinetic profile of IRBM-Z-2 following 100 mpk (PO and IP QD) dosing. IRBM-Z-2 was administered to C57BL/6 mice (n=3 per group) at 100 mg/kg QD by intraperitoneal (IP) and oral (PO) routes. Single IP administration showed plasma concentrations exceeded the calculated EC_{99} (1.39 μM, based on ZIKV EC_{50} and adjusted for 10% FBS and unbound fraction) for over 24 hours. Single PO dose (at 100 mg/kg) maintained plasma exposure above the EC_{99} up to 20 hours.

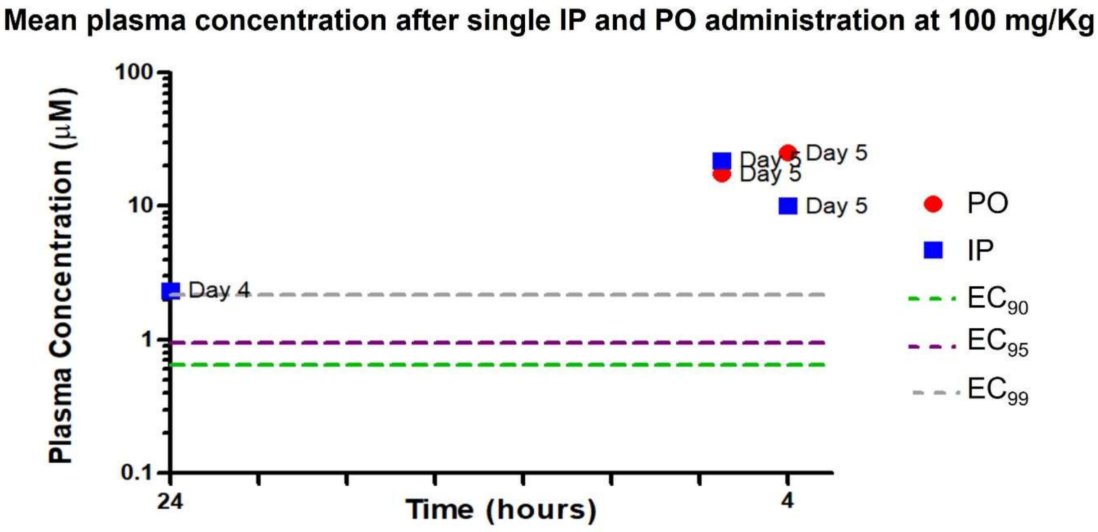

**Route**	**Dose** (mpk)	**Matrix**	**Time**	$\mathbf{C_{total}}$ (μM)
IP	100 QD	Plasma	4 days-24 h	2.32
			5 days-1 h	22.1
			5 days-4 h	10.0
PO			4 days-24 h	BLQ
			5 days-1 h	17.4
			5 days-4 h	25.1

BLQ = below the limit of quantification

Supplementary Figure 15 | IP and PO repeated administration of compound IRBM-Z-2 at 100 mg/kg, once a day for 5 days. Tolerability and pharmacokinetic consistency studies. IRBM-Z-2 was dosed daily for 5 consecutive days at 100 mg/kg in C57BL/6 mice (n=3 per group) by intraperitoneal and oral route. The compound was well tolerated by both routes, and no clinical signs or weight loss were observed. The graph and table above represent the IRBM-Z-2 plasma concentrations measured at day 4 and 5. Plasma concentrations during the 5-day dosing period were consistent with those observed in the single dose studies. The high C_{max} and plasma exposure values observed supported the selection of IRBM-Z-2 for evaluation in a ZIKV infection mouse model.

.

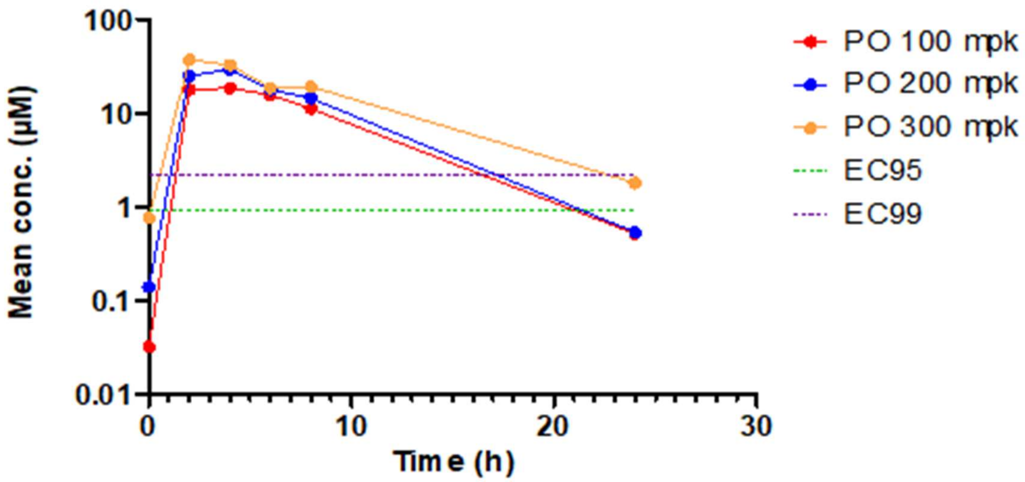

Route	Dose (mpk)	Matrix	C_{max} (µM)	T_{max} (h)	AUC (µM*Hours)	C_{trough} (µM)
PO	100 QD	Plasma	23.3	4	208	0.518
	200 QD		29.2	4	278	0.539
	300 QD		38.1	3	364	1.81

Supplementary Figure 16 | Pharmacokinetic profile of compound IRBM-Z-2 after repeated oral administration to C57BL/6 mice at 100, 200 and 300 mg/kg, once a day for 7 days. Maximum tolerated dose (MTD) study conducted in C57BL/6 mice (15 animals, n=5 per group). IRBM-Z-2 was orally administered at 3 concentrations (100 mg/kg, 200 mg/kg and 300 mg/kg) once a day for 7 days. IRBM-Z-2 was well tolerated at all doses with no clinical signs of toxicity or significant weight loss observed. The graph and table above represent the IRBM-Z-2 plasma concentrations (for all three doses) measured at differing time points on day 9. Compound plasma concentration remained above the EC_{99} threshold for at least 20 hours in all dose groups. The 100 mg/kg dose was selected for further studies**.**

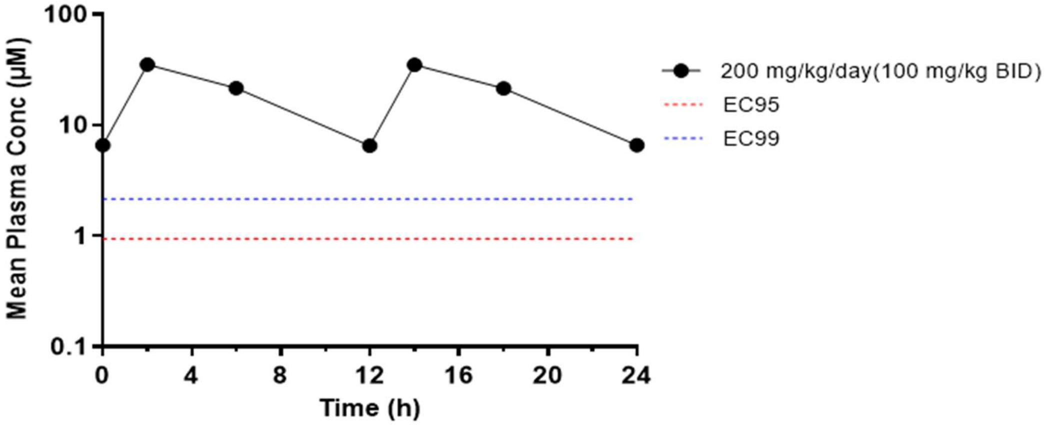

**Route**	**Dose** (mpk)	**Matrix**	**Estimated** C_{max} (µM)	**Estimated** T_{max} (h)	**AUC** (µM*Hours)	C_{trough} (µM)
PO	100 BID	Plasma	35	2	478	6.5

Supplementary Figure 17 | PK profile of IRBM-Z-2 after repeated oral administration to C57BL/6 mice at 100 mg/kg BID. Single day of BID administration to C57BL/6 mice (n=3) shows plasma IRBM-Z-2 concentrations above the EC_{99} for the full 24-hour period.

Group	N	Weights and Clinical Observations	Treatment (IP)		Challenge (IP)	In-Life Blood Collection (SST)	Terminal Blood Collection (SST)
1	2	Daily weights and BID observations<br><br>Weights on blood collection and sedation days, and as needed for treatments.	Vehicle	SD 0 (1 h p.i.)<br>SD 1 (25 h p.i.)<br>SD 2 (49 h p.i.)<br>SD 3 (73 h p.i.)<br>SD 4 (97 h p.i.)	SD 0<br>ZIKV	d.p.i 1, 4	Max bleed at termination: d.p.i 5 (120 h p.i.)
2	2					d.p.i 2	
3	2					d.p.i 3	
4	3		Drug<br>100<br>mpk			d.p.i 1, 4	
5	3					d.p.i 2	
6	3					d.p.i 3	

d.p.i = days post infection

Supplementary Table 5 | IP 5-Day PoC study design.

Group	N	Weights and Clinical Observations	Treatment (PO)		Challenge (IP)	In-Life Blood Collection (SST)	Terminal Blood Collection (SST)
1	3	Daily weights and BID observations<br><br>Weights on blood collection and sedation days, and as needed for treatments.	Vehicle	SD 0 (1 h p.i.)<br>SD 0 (13 h p.i.)<br>SD 1 (AM)	SD 0<br>ZIKV	d.p.i 5, 11	N/A
2	3					d.p.i 1, 7	
3	3					d.p.i 3, 9	
4	3		Drug<br>100<br>mpk	SD 0 (1 h p.i.)<br>SD 0 (13 h p.i.)<br>BID SD 1-13<br>(12 h timepoint)		d.p.i 5, 11	Max bleed at termination: d.p.i 14
5	3					d.p.i 1, 7	
6	3					d.p.i 3, 9	

d.p.i = days post infection

Supplementary Table 6 | PO 14-Day Survival and efficacy study design.

a

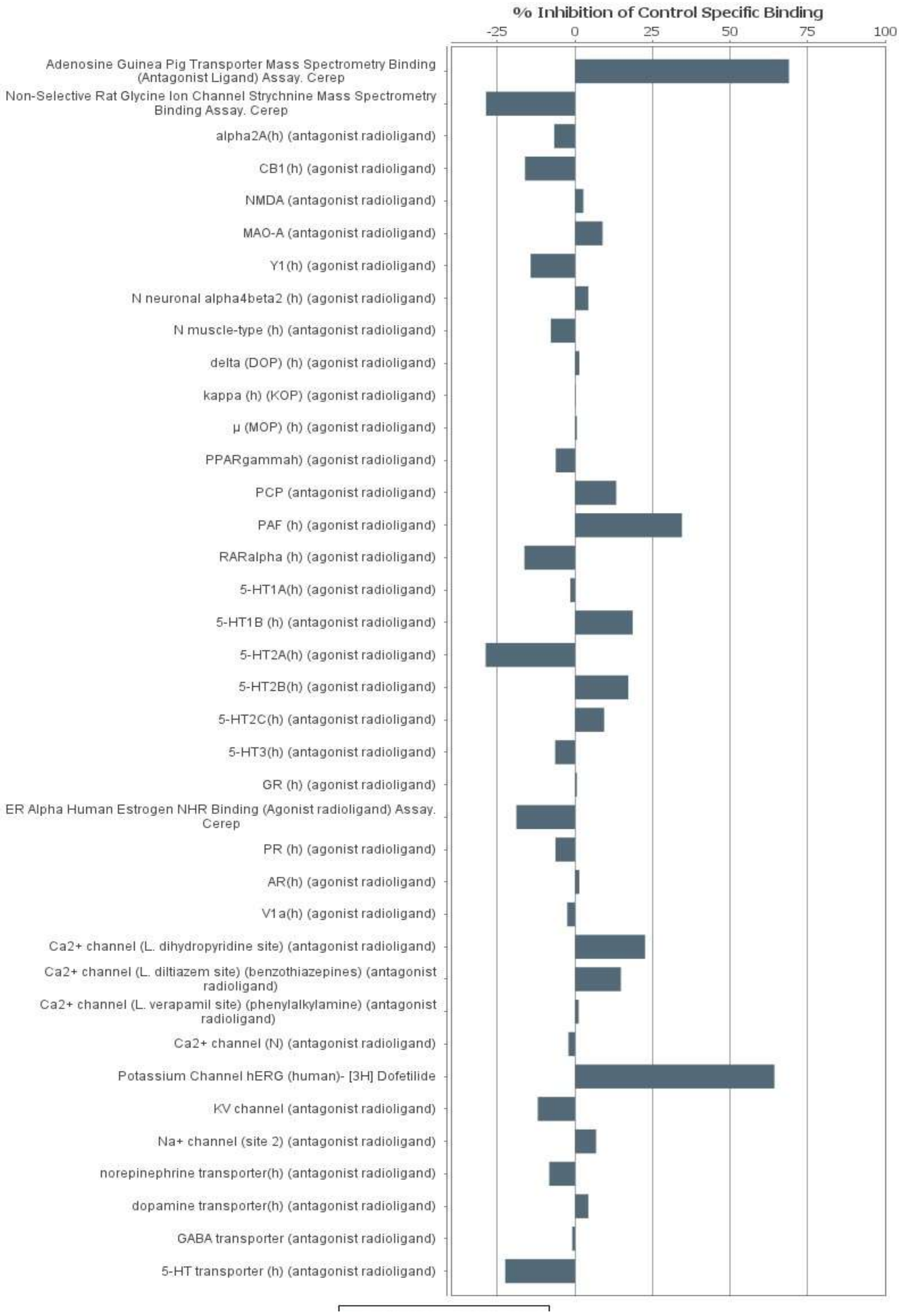
% Inhibition of Control Specific Binding
-25
0
25
50
75
100
Adenosine Guinea Pig Transporter Mass Spectrometry Binding (Antagonist Ligand) Assay. Cerep
Non-Selective Rat Glycine Ion Channel Strychnine Mass Spectrometry Binding Assay. Cerep
alpha2A(h) (antagonist radioligand)
CB1(h) (agonist radioligand)
NMDA (antagonist radioligand)
MAO-A (antagonist radioligand)
Y1(h) (agonist radioligand)
N neuronal alpha4beta2 (h) (agonist radioligand)
N muscle-type (h) (antagonist radioligand)
delta (DOP) (h) (agonist radioligand)
kappa (h) (KOP) (agonist radioligand)
μ (MOP) (h) (agonist radioligand)
PPARgammah) (agonist radioligand)
PCP (antagonist radioligand)
PAF (h) (agonist radioligand)
RARalpha (h) (agonist radioligand)
5-HT1A(h) (agonist radioligand)
5-HT1B (h) (antagonist radioligand)
5-HT2A(h) (agonist radioligand)
5-HT2B(h) (agonist radioligand)
5-HT2C(h) (antagonist radioligand)
5-HT3(h) (antagonist radioligand)
GR (h) (agonist radioligand)
ER Alpha Human Estrogen NHR Binding (Agonist radioligand) Assay. Cerep
PR (h) (agonist radioligand)
AR(h) (agonist radioligand)
V1a(h) (agonist radioligand)
Ca2+ channel (L. dihydropyridine site) (antagonist radioligand)
Ca2+ channel (L. diltiazem site) (benzothiazepines) (antagonist radioligand)
Ca2+ channel (L. verapamil site) (phenylalkylamine) (antagonist radioligand)
Ca2+ channel (N) (antagonist radioligand)
Potassium Channel hERG (human)- [3H] Dofetilide
KV channel (antagonist radioligand)
Na+ channel (site 2) (antagonist radioligand)
norepinephrine transporter(h) (antagonist radioligand)
dopamine transporter(h) (antagonist radioligand)
GABA transporter (antagonist radioligand)
5-HT transporter (h) (antagonist radioligand)

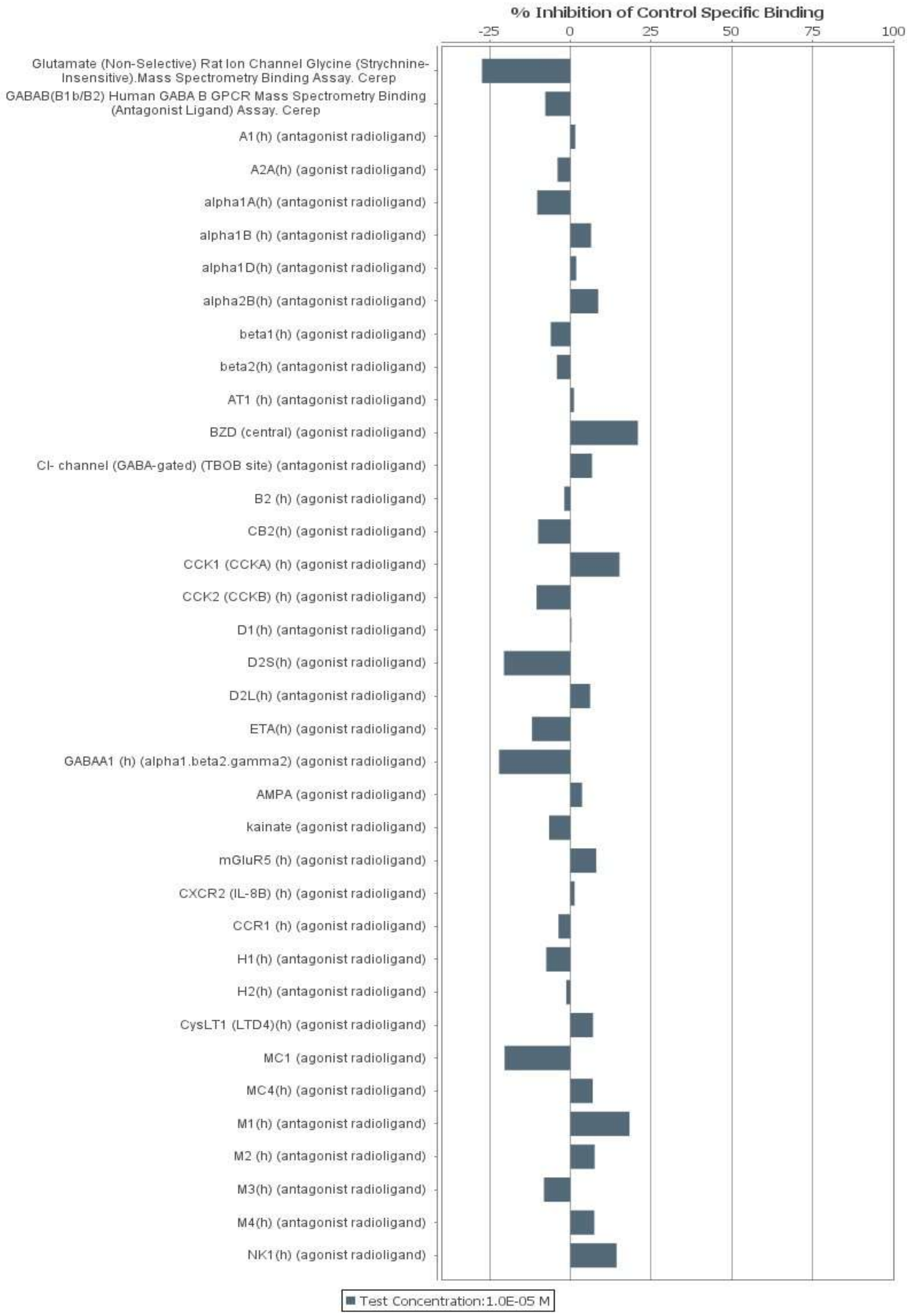

b

**ASSAY**	**Compound**	**% Inh.** (10 μM)
Potassium Channel hERG (human)-[3H] Dofetilide	IRBM-Z-2	64.2
Adenosine Guinea Pig Transporter Mass Spectrometry Binding (Antagonist Ligand) Assay, Cerep		69.0

Supplementary Figure 18 | Preclinical safety panel for IRBM-Z-2. A safety pharmacology panel comprising 87 receptor, ion channel and enzyme assays was performed by Eurofins Cerep (France), using the methodologies described on their website

(https://www.eurofinsdiscovery.com/catalog/safetyscreen87-panel-fr/P342). IRBM-Z-2 was tested at a concentration of 10 µM. Target binding was assessed by measuring the percentage inhibition of a reference ligand's binding to each target, while enzyme inhibition was determined by the percent inhibition of control enzymatic activity. **a** Histogram of IRBM-Z-2 preclinical safety panel **b** Results showing inhibition or stimulation higher than 50%.

Supplementary Methods

Kinetic solubility determination

Kinetic solubility of the compounds was measured in phosphate buffer saline at pH 7.4 and dimethyl sulfoxide (DMSO) (196 µL), placed in a 96-deepwell plate according to the number of test compounds. 4 µL of test compound stock solution (10 mM in DMSO) were spiked in both DMSO and aqueous medium wells to obtain a final concentration of 200 µM, in two replicates. The plate was shaken for 2 hours at 25 °C. After centrifugation (15 min, 4500 rpm, 25 °C), the supernatant (150 µL) was directly analysed by ACQUITY UPLC I-Class equipped with a Diode Array Detector (DAD) working in a range between 210 nm and 400 nm. Data analysis was performed with Empower 3.0 (Waters). The kinetic solubility values were calculated by comparing the chromatographic peak area in buffer to the corresponding peak area in DMSO. Results which are below the reliable limit of the assay are shown as < 5 µM.

PAMPA permeability assay

Passive permeability of the compounds was assessed in a PAMPA assay using a 96-well multiscreen filter plate (Gentest™ Pre-coated PAMPA Plate System, Corning) with LC-MS/MS detection. Up to 4 test compounds were pooled according to their m/z ratio and retention time to get a final concentration of 2.5 mM in DMSO for each compound. The PAMPA Plate System (Corning Gentest™ Pre-coated), stored at -20 °C was kept at room temperature (rt) for one hour before starting the incubation. Working control and test compound solutions were dispensed in five replicates using Tecan D300e into the donor compartment, then 300 µL PBS were added to yield a final test compound concentration of 5 µM. 200 µL of PBS were added to each well of the acceptor compartment (pre-coated filter). For the equilibrium samples used for mass balance, working solutions were dispensed using Tecan D300e in a 96-well plate, then 500 µL of PBS were added to yield a final test compound concentration of 3 µM. The donor compartment was coupled with the acceptor compartment and the plate assembly incubated at rt, then gently stirred at 450 rpm. After 5 h, 50 µL were transferred from each well into a new 96-well plate, also including the equilibrium samples. 50 µL of ISWS (Labetalol 200 nM, Diclofenac 200 nM, Imipramine 200 nM in acetonitrile + 0.1% formic acid) were added to each well, then the plate was centrifuged (15 min, 4500 rpm, 25 °C) prior to LC-HRMS analysis. Prior to starting with data analysis, permeability controls (Ranitidine, Carbamazepine) were investigated to decide if some replicates need to be excluded from the calculation. The peak area ratios of compound to the internal standard were used to directly calculate the permeability, expressed as Pe units: $\times 10^{-6}$ cm/s.

MDCK permeability assay

The apparent permeability of compounds was determined by Eurofins using their standard MDCK Protocol. The apparent permeability coefficient (Papp) of the test compound was calculated as follows:

$$P_{app}(cm/s) = \frac{V_R * C_{R,end}}{\Delta t} * \frac{1}{A * (C_{D,mid} - C_{R,mid})}$$

where VR is the volume of the receiver chamber. $C_{R,end}$ is the concentration of the test compound in the receiver chamber at the end time point, Δt is the incubation time and A is the surface area of the cell monolayer. $C_{D,mid}$ is the calculated mid-point concentration of the test compound in the donor compartment, which is the mean value of the donor concentration at time = 0 minutes and the donor concentration at the end time point. $C_{R,mid}$ is the mid-point concentration of the test compound in the receiver compartment, which is one half of the receiver concentration at the end time point. Concentrations of the test compound were expressed as peak areas of the test compound.

Recovery of the test compound from the permeability assay

The recovery of the test compound was calculated as follows:

$$\text{Recovery(\%)} = \frac{V_D * C_{D,end} + V_R * C_{R,end}}{V_D * C_{D0}} * 100$$

where VD and VR are the volumes of the donor and receiver chambers, respectively. $C_{D,end}$ is the concentration of the test compound in the donor sample at the end time point. $C_{R,end}$ is the concentration of the test compound in the receiver sample at the end time point. C_{D0} is the concentration of the test compound in the donor sample at time zero. Concentrations of the test compound are expressed as peak areas of the test compound. Fluorescein was used as the cell monolayer integrity marker. The fluorescein permeability assessment (in the A-B direction at pH 7.4 on both sides) was performed after the permeability assay for the test compound. The cell monolayer that had a fluorescein permeability of less than 1.5×10^{-6} cm/s for Caco-2 and MDR1-MDCKII cells and 2.5×10^{-6} cm/s for MDCKII cells was considered intact, and the permeability result of the test compound from the intact cell monolayer is reported.

Microsomal stability intrinsic clearance determination

Human, rat (male Sprague–Dawley) and mouse (C57BL/6) liver microsomes (LM) were purchased from BioIVT. Dehydrogenase, NADPH-generating system (1 mM NADP, 2.5 mM glucose 6-phosphate and 2 U/ml glucose 6- phosphate dehydrogenase) and $MgCl_2$ were purchased from Sigma Aldrich. Assays were performed in the presence of 0.25 mg/ml of microsomal protein, with an NADPH-generating system (1 mM NADP, 2.5 mM glucose 6-phosphate and 2 U/ml glucose 6-phosphate dehydrogenase) in 100 mM potassium phosphate buffer containing 3.3 mM $MgCl_2$ (pH7.4). In metabolic stability assays, with microsomes from various livers, compound A was diluted into the incubation mixture from a 10 mM stock solution in DMSO and incubated at a final concentration of 0.3 μM (final DMSO concentration in incubation 0.1%). Microsomes and substrate were preincubated at 37 °C for 5 min and then the enzymatic reactions were initiated by the addition of cofactors and incubated at 37 °C for up to 60 min. Parent compound degradation was monitored over a 60-minute incubation period, with samples collected at 8 time points: 0, 5, 10, 15, 20, 30, 45 and 60 minutes. Incubations were terminated by the addition of an equal volume of acetonitrile containing the internal standard (I.S.). Control incubations, in which the substrate was incubated in the same buffer system with LM but without the NADPH-generating system, were also performed. Samples were centrifuged at 14,000 rcf for 15 min, and the supernatants were analyzed using a Dionex Ultimate 3000 RS system coupled to a Q-Exactive Orbitrap high-resolution mass spectrometer (Thermo Scientific),

operating in full scan mode for intrinsic clearance determination. Raw files were acquired through Thermo Xcalibur™ 3.1 (Thermo Fisher).

Hepatocyte stability studies

Human, rat and dog hepatocyte-fibroblast co-cultures (HepatoPac from BioIVT) were maintained according to the manufacturer's instructions. Prior to the addition of test compounds, the cultures were washed once with supplemented application medium and incubated in a 37 °C, 5% CO_2 incubator for 2 h to allow cells to adapt to serum-free conditions. The test compound was dissolved in DMSO and incubated at a final assay concentration of 3 μM. Warfarin and ketoprofen were used as low-clearance control compounds for human, rat and dog, respectively. Incubations containing only stromal cells served as a control to assess hepatocyte-mediated metabolism up to 96 h post-dosing. At the end of the incubation period, two volumes of quenching solution (acetonitrile and 0.1% formic acid containing 100 mM internal standards), were added to one volume of cell suspension. Collected samples were kept on an orbital shaker for 20 min prior to the centrifugation step. An aliquot of clean supernatant was dried under liquid nitrogen flow and stored at -20 °C. Samples were reconstituted with 50:50 water/acetonitrile containing 0.1% formic acid prior to LC-MS analysis using high resolution mass spectrometry (HRMS). Data analysis was performed by calculating the slope of the Ln (% remaining) versus time plot to obtain the half-life ($t_{1/2}$), which was then used to calculate the intrinsic clearance (CL_{int}) expressed as μL/min/million cells.

***In Vitro* CYP inhibition assay**

The P450-Glo CYP1A2 Screening System (V9770), P450-Glo CYP2D6 Screening System (V9890) and P450-Glo CYP3A4 Screening System (V9910) were obtained from Promega Corporation. Each screening system was used according to the manufacturer's guidelines. The test compound and the specific CYP inhibitor for each isoform were dispensed in 384-well microtiter plates using Tecan D300e to make titration curves in duplicates (11 points, 1:2 dilution). All reactions contained a final DMSO concentration of 0.3%. Furafylline, quinidine and ketoconazole were used as known inhibitors of CYP1A2, CYP2D6 and CYP3A4, respectively. A 4X enzyme reaction mixture containing the specific CYP membranes was prepared for each of the three CYP isoforms. Negative control reactions were prepared using control membranes lacking CYP activity. An appropriate volume of each reaction mixture was added to the wells, and a 10-minute pre-incubation step was performed at 37 °C under shaking at low speed. A 2X NADPH regeneration system containing solution A and solution B was prepared according to vendor guidelines and added to each well to initiate the CYP reactions. Incubations were carried out at 37 °C under shaking at low speed, for the following time intervals: CYP1A2 - 10min; CYP3A4 - 15min; CYP2D6 - 45min. At the end of the incubation step, CYP reactions were stopped by adding the appropriate volume of reconstituted luciferin detection reagent specific to each CYP isoform. Samples were incubated at rt for 20 min prior to luminescence measurement. An IC_{50} value for the test compound and known inhibitors was determined through nonlinear regression curve fitting analysis, using XLFit software and the sigmoidal dose-response curve equation.

In Vitro human hepatocyte CYP induction assay

Cryopreserved human hepatocytes were thawed quickly into a 37 °C water bath and resuspended in InVitroGRO CP Medium supplemented with a TORPEDO antibiotic mix. Hepatocytes were plated in collagen-coated 96-well plates at a seeding density of 0.6 x 10^6cells/mL and left in the incubator at 37 °C, 5% CO_2. After 4 h, the medium was replaced with InVitroGRO HI Medium supplemented with a TORPEDO antibiotic mix and hepatocytes were returned to the incubator at 37 °C, 5% CO_2 until the following day. Cell morphology was monitored to confirm monolayer formation prior to starting the induction assay. The test compound and positive controls were dissolved in DMSO to obtain a 1000-fold stock concentration. On Day 2, dosing solutions were prepared in the culture medium at various concentrations and added to each well in triplicates (0.1% final DMSO concentration in assay). The vehicle control was prepared with 0.1% DMSO. Omeprazole was used as a positive control inducer for CYP1A2 while rifampicin was used as a positive control inducer for CYP3A4 and CYP2D6. After 24 h, old incubation medium was removed and replaced with fresh dosing solution containing the test compound or the positive control inducer. 48 h after treatment, culture medium was removed, cells were washed twice with phosphate-buffered saline (PBS) and harvested with RLT lysis buffer (Qiagen) containing β-mercaptoethanol. RNA isolation was performed on a Hamilton liquid handler using the Macherey-Nagel isolation kit according to the manufacturer's protocol. Reverse transcription was performed with the QuantiTect® Probe RT-PCR Kit (Qiagen) and Taqman probes for CYP1A2, CYP2D6, CYP3A4 and GAPDH were used for qTR-PCR analysis. Relative quantification measured change relative to the vehicle control and normalized to the endogenous control GAPDH for CYP1A2, CYP2D6 and CYP3A4 mRNA expression.

hERG polarization assay

Compounds were dissolved in 100% DMSO, and their ability to compete with E-4031 and block the hERG channel was evaluated using a final titration curve ranging from 30,000 nM to 0.5 nM, in biological duplicates (1:3 dilutions), using the Predictor™ hERG Fluorescence Polarization Assay Kit (Life Technologies, Burlington, ON, Canada). E-4031 and Cisapride were used as positive controls. The assay was performed in a 384-well format, and all measurements were made according to the manufacturer's instructions. Fluorescence polarization was measured using a Tecan ULTRA microplate reader equipped with a fluorescence polarization module. The results were expressed as percent inhibition, and IC_{50} values were determined in cases in which significant hERG channel inhibition was observed. IC_{50} values were calculated from dose-response curves by nonlinear regression using XLfit 4.2 (IDBS Ltd), applying the 4-parameter logistic model.

Plasma protein binding determination

Verapamil, Carbamazepine and Warfarin were purchased from Sigma Aldrich. Plasma matrixes were pooled from four healthy subjects and obtained from CliniSciences. The Phosphate Buffer Solution (PBS; pH 7.4) used was of analytical grade and had a proven pH. Stock solution aliquots of test and control compounds were prepared separately in DMSO at a concentration of 10 mM. Compounds were pooled to a final working solution (WS) of 200 µM in DMSO. Samples were prepared by mixing the WS with plasma to achieve a final concentration of 1 µM. The incubation

plate (RED device) was assembled by placing 200 uL of pool solution in plasma into the sample chamber (red ring) and 350 uL of phosphate buffer solution pH 7.4 (0.1 M phosphate buffer + 0.15 M sodium chloride) into the corresponding buffer chamber for each replicate, respectively. The plate was covered with sealing tape and incubated at 37 °C under 5% CO_2 on an orbital shaker at 300 rpm for 4 h. An aliquot of the remaining plasma samples (T0 pre-dialysis) was used to calculate the Recovery and was immediately quenched after incubation plate assembly. At the end of the incubation period, 50 µL from both the buffer and plasma chambers were removed and placed in separate tubes, and a matrix mixing approach was used (50 µL of blank plasma were added to 50 µl of the buffer samples, and 50 µl of phosphate buffer pH 7.4 were added to 50 µL of the collected plasma samples). After that, 200 µL of ACN were added to each sample to precipitate protein and release compound. Samples were vortexed, then centrifuged at + 4 °C for 40 min at 140.000 rpm. Supernatants (150 uL) were transferred to a 96-well analysis plate. The plate was dried under N_2 pressure. Then, samples were reconstituted with the appropriate solvent containing 0.1 µM IS for the analysis. LC-MRM analyses were carried out on various LC/MS instruments, all of which were equipped with an Electrospray (ESI) ion source. The mass spectrometer was directly connected to the liquid chromatograph, equipped with a Sample Manager. The LC-MRM conditions varied, depending on the characteristics of the compounds to be tested. Recovery was found to be between 75% and 100% for all replicates. The free fraction was calculated as the ratio of compound concentration in the buffer chamber to that in the plasma chamber. The fraction of unbound drug (% f_u) and recovery were calculated based on the following formula: % f_u = (Area Ratiobuffer *V_{buffer}/Area Ratioplasma*V_{plasma}) x 100.

Synthesis of IRBM-Z-1, *R*-(+)-IRBM-Z-1 and IRBM-Z-2

General experimental details

Solvents and reagents were obtained from commercial suppliers and were used without further purification. Silica gel chromatography purifications were performed on prepacked cartridges using a Biotage system. UPLC-MS analyses were performed on a Waters™ Acquity UPLC, equipped with a diode array and a ZQ mass spectrometer, using an X-Terra C18 column (5 µm, 4.6 x 50 mm) or a BEH C18 column (1.7 µm, 2.1 x 50 mm). The mobile phase comprised a linear gradient of binary mixtures of H_2O (A) and MeCN (B), each containing 0.1% formic acid. The linear gradient used was: (A): 90% (0.1 min), 90-0% (2.6 min), 0% (0.3 min), 0-90% (0.1 min) with a 0.5 mL/min flow. ^1H, ^{19}F, ^{13}C NMR spectra were performed at 400, 377 and 101 MHz on a 400 MHz Bruker spectrometer. Chemical shifts (δ) were reported in parts per million, downfield to tetramethylsilane, using DMSO-d_6 as a solvent unless otherwise noted. Coupling constants (*J*) were reported in Hertz (Hz). Multiplicities were reported as singlet (s), broad (br), doublet (d), doublet of doublets (dd), doublet of doublets of doublets (ddd), triplet (t), doublet of triplet (dt) or multiplet (m). Unless indicated, spectra were acquired at 300 K. Temperatures were expressed in degrees Celsius (°C) and were uncorrected. The reported yields were the actual isolated yields of purified material and were not optimized. Where the synthesis of intermediates and starting materials was not described, the compounds were either commercially available or prepared from commercially available materials using standard methods. High resolution molecular ion determinations (HRMS) were performed using a Dionex Ultimate 3000 RS UHPLC system coupled to an Orbitrap™ Q-Exactive high-resolution mass spectrometer (Thermo Scientific) operating in ESI positive full scan (m/z range 100-1000e at resolution 140.000 FWHM at 200 m/z). Mass error was within 2.2 ppm accuracy. Low resolution molecular ion determinations (LRMS) of intermediates and final compounds IRBM-Z-1, *R*-(+)- IRBM-Z-1 and IRBM-Z-2, were performed using a Waters™ Acquity UPLC, equipped with a diode array and a ZQ mass spectrometer with a BEH C18 column (1.7 µm, 2.1 x 50 mm).

(3-(1-Phenylpropan-2-yl)-1,2,3-oxadiazol-3-ium-5-yl)((3-(trifluoromethyl)phenyl)-carbamoyl)amide (IRBM-Z-1)

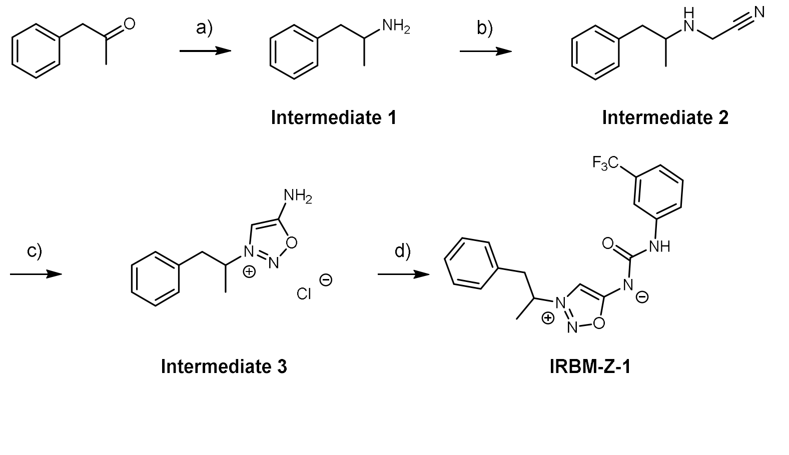

Supplementary Scheme 1 | Synthetic pathway for **IRBM-Z-1**: a) Pd/C NH_4HCO_2, H_2O/MeOH, rt, 16 h (58%); b) 2-bromoacetonitrile, DIPEA, MeCN, rt, 16 h (86%); c) 1. *t*-Butyl nitrite, THF, rt, 4 h; 2. HCl

(4N), dioxane, rt (82% overall 2 steps); d) 3-(trifluoromethyl)benzoic acid, TEA, DPPA, toluene, 80 °C, 2 h (87%).

Step 1: 1-Phenylpropan-2-amine (Intermediate 1).
Ammonium formate (24.44 g, 387.54 mmol) was dissolved in a mixture of water (9 mL) and methanol (80 mL), and the solution was treated with 1-phenylpropan-2-one (4.95 mL, 37.26 mmol) and Pd/C (10% w/w) (1.19 g, 11.18 mmol). The mixture was stirred at rt for 16 h then filtered through a pad of Celite and washed with methanol. The filtrate was evaporated under reduced pressure to get a white powder which was suspended in EtOAc (150 mL) and stirred at rt for 1 h. The solid formed was isolated by filtration to get the desired product as a salt (4.2 g). 500 mg of isolated product were dissolved in methanol (1.5 mL) and charged on a SCX cartridge (5 g). The resin was washed with methanol followed by a 2 M NH_3 solution in methanol. After removal of the solvents under reduced pressure the title compound was obtained as a white powder (350 mg, 58%). ^{1}H NMR (400 MHz, DMSO-d_6): δ 7.30-7.26 (m, 2H), 7.20-7.17 (m, 3H), 3.03-2.96 (m, 1H), 2.55-2.48 (m, 2H), 0.95 (d, J = 4.0 Hz, 3H); LCMS (ES$^+$): m/z calculated for $C_9H_{13}N$ 135.10, found 136 [M+H]$^+$; HPLC t_R = 0.59 min.

Step 2: 2-((1-Phenylpropan-2-yl)amino)acetonitrile (Intermediate 2).
A suspension of 1-phenylpropan-2-amine (**Intermediate 1**) (1.31 g, 9.71 mmol) and DIPEA (3.44 mL, 19.42 mmol) in MeCN (11.8 mL) was treated with 2-bromoacetonitrile (0.67 mL, 9.7 mmol) and the reaction mixture was stirred at rt for 16 h. Solvent was then removed under reduced pressure and the residue treated with water (10 mL) and extracted with EtOAc (3 x 50 mL). The collected organics were washed with brine, dried over Na_2SO_4 and evaporated under reduced pressure to get the title compound as a colorless oil (1.45 g, 86%). ^{1}H NMR (400 MHz, DMSO-d_6): δ 7.32-7.28 (m, 2H), 7.22-7.18 (m, 3H), 3.73-3.61 (m, 2H), 2.95-2.90 (m, 1H), 2.77 (dd, J_1 = 4.0 Hz, J_2 = 12.0 Hz, 1H), 2.46-2.41 (m, 2H), 0.92 (d, J = 4.0 Hz, 3H); LCMS (ES$^+$): m/z calculated for $C_{11}H_{14}N_2$ 174.12, found 175 [M+H]$^+$; HPLC t_R = 0.81 min.

Step 3: 5-Amino-3-(1-phenylpropan-2-yl)-1,2,3-oxadiazol-3-ium chloride (Intermediate 3).
A solution of 2-((1-phenylpropan-2-yl)amino)acetonitrile (**Intermediate 2**) (1.4 g, 8.0 mmol) in THF (3.9 mL) was treated with *tert*-butyl nitrite (3.8 mL, 32.1 mmol). The solution was stirred at rt for 4 h. Then, 4 N HCl in dioxane (13.1 mL, 52.2 mmol) was added, and the reaction mixture was stirred at rt for 18 h. Diethyl ether was added to the mixture, and the precipitate formed was collected by filtration and washed with diethyl ether to afford the title compound as a white powder (1.39 g, 82%). ^{1}H NMR (400 MHz, DMSO-d_6): δ 9.71 (s, 2H), 8.20 (s, 1H), 7.34-7.21 (m, 5H), 5.33-5.27 (m, 1H), 3.32-3.30 (m, 2H), 1.65 (d, J = 4.0 Hz, 3H); LCMS (ES$^+$): m/z calculated for $C_{11}H_{14}N_3O^+$ 204.11, found 204 [M]$^+$; HPLC t_R = 0.84 min.

Step 4: (3-(1-Phenylpropan-2-yl)-1,2,3-oxadiazol-3-ium-5-yl)((3-(trifluoromethyl)phenyl) carbamoyl)amide (IRBM-Z-1).
A solution of 3-(trifluoromethyl)benzoic acid (277.6 mg, 1.5 mmol) in toluene (1.7 mL, 0.02 mol) was treated with TEA (0.2 mL, 1.6 mmol) and DPPA (0.3 mL, 1.6 mmol). The mixture was heated at 70 °C for 1 h then **Intermediate 3** (175 mg, 0.7 mmol) was added and the mixture stirred at 80 °C for 2 h. The mixture was cooled to rt and quenched with the addition of 1 N

$NaHCO_3$ (aq. sol.) and extracted with EtOAc. The combined organic phase was washed with water and brine, dried over Na_2SO_4 and evaporated under reduced pressure. The residue obtained was triturated with petroleum ether to get the title compound as a light-yellow solid (258 mg, 87%). ^{1}H NMR (400 MHz, DMSO-d_6): δ 9.65 (s, 1H), 8.32 (s, 1H), 8.27 (br s, 1H), 7.68 (br d, J = 8.0 Hz, 1H), 7.43 (t, J = 8.0 Hz, 1H), 7.31-7.27 (m, 2H), 7.24-7.19 (m, 4H), 5.16-5.11 (m, 1H), 3.32 (m, 2H), 1.65 (d, J = 8.0 Hz, 3H); ^{19}F NMR (377 MHz, DMSO-d_6): δ -61.28 (s, 3F); LCMS (ES$^+$): m/z calculated for $C_{19}H_{17}F_3N_4O_2$ 390.13; found 391 $[M+H]^+$; HPLC t_R = 2.0 min; HRMS (m/z): $[M+H]^+$ calculated for $C_{19}H_{17}F_3N_4O_2$, 391.13764; found 391.13770.

***R*-(+)-(3-(1-Phenylpropan-2-yl)-1,2,3-oxadiazol-3-ium-5-yl)((3-(trifluoromethyl) phenyl)-carbamoyl)amide (*R*-(+)-IRBM-Z-1)**

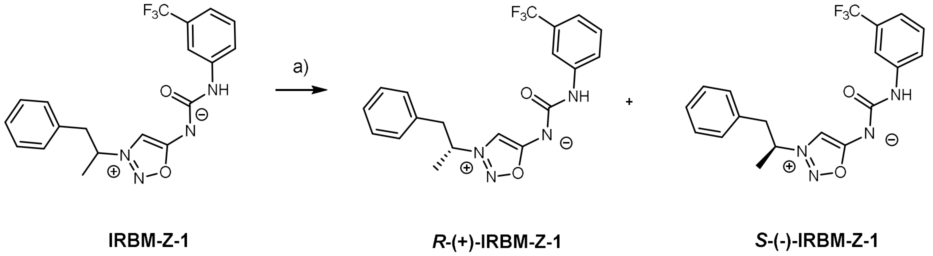

Supplementary Scheme 2 | Synthetic pathway for ***R*-(+)-IRBM-Z-1** and ***S*-(-)-IRBM-Z-1**: a) SFC enantiomeric separation: compound ***R*-(+)-IRBM-Z-1** (23%), compound ***S*-(-)-IRBM-Z-1** (24%).

(*R*)-(3-(1-Phenylpropan-2-yl)-1,2,3-oxadiazol-3-ium-5-yl)((3-(trifluoromethyl)phenyl)-carbamoyl)amide (*R*-(+)-IRBM-Z-1) and (*S*)-(3-(1-phenylpropan-2-yl)-1,2,3-oxadiazol-3-ium-5-yl)((3-(trifluoromethyl)phenyl)carbamoyl)amide (*S*-(-)-IRBM-Z-1).

(3-(1-Phenylpropan-2-yl)-1,2,3-oxadiazol-3-ium-5-yl)((3-(trifluoromethyl)phenyl)carbamoyl) amide (**IRBM-Z-1**) (45 mg, 0.12 mmol) was subjected to SFC purification (Column: Chiralpak IA (cellulose) - Run time 19 min - Eluent: MeOH – Method: 15% MeOH (2 min), 15-20% MeOH (8 min), 20% MeOH (7 min), 20-15% MeOH (1 min), 15% MeOH (1 min) to get (*R*)-(3-(1-phenylpropan-2-yl)-1,2,3-oxadiazol-3-ium-5-yl)((3-(trifluoromethyl)phenyl)carbamoyl)amide (1st eluted compound) as a white powder (10.2 mg, 23%). $[\alpha]^{20}_D$= +196.75 (0.161 M in CH_3CN); ^{1}H NMR (400 MHz, DMSO-d_6): δ 9.65 (s, 1H), 8.32 (s, 1H), 8.27 (br s, 1H), 7.68 (br d, J = 8.0 Hz, 1H), 7.43 (t, J = 8.0 Hz, 1H), 7.31-7.27 (m, 2H), 7.24-7.19 (m, 4H), 5.16-5.11 (m, 1H), 3.32 (m, 2H), 1.65 (d, J = 8.0 Hz, 3H); ^{13}C NMR (101 MHz, DMSO-d_6): δ 172.6, 159.0, 141.9, 136.0, 129.4, 129.4, 129.1, 128.8, 128.5, 127.0, 125.7, 123.0, 121.3, 117.2, 113.6, 102.8, 62.8, 40.5, 19.3. LCMS (ES$^+$): m/z calculated for $C_{19}H_{17}F_3N_4O_2$ 390.13; found 391 $[M+H]^+$; HPLC t_R = 2.0 min; HRMS (m/z): $[M+H]^+$ calculated for $C_{19}H_{17}F_3N_4O_2$, 391.13764; found 391.13776. And (*S*)-(3-(1-phenylpropan-2-yl)-1,2,3-oxadiazol-3-ium-5-yl)((3-(trifluoromethyl)phenyl)-carbamoyl)-amide (2nd eluted compound) as a white powder (10.7 mg, 24%). $[\alpha]^{20}_D$= -183.11 (0.161 M in CH_3CN); ^{1}H NMR (400 MHz, DMSO-d_6): δ 9.65 (s, 1H), 8.32 (s, 1H), 8.27 (br s, 1H), 7.68 (br d, J = 8.0 Hz, 1H), 7.43 (t, J = 8.0 Hz, 1H), 7.31-7.27 (m, 2H), 7.24-7.19 (m, 4H), 5.16-5.11 (m, 1H), 3.32 (m, 2H), 1.65 (d, J = 8.0 Hz, 3H); LCMS (ES$^+$): m/z calculated for $C_{19}H_{17}F_3N_4O_2$ 390.13; found 391 $[M+H]^+$; HPLC t_R = 2.0 min; HRMS (m/z): $[M+H]^+$ calculated for$C_{19}H_{17}F_3N_4O_2$, 391.13764; found 391.13770.

(3-((5-(4,6-Dimethylpyrimidin-5-yl)pyridin-2-yl)methyl)-1,2,3-oxadiazol-3-ium-5-yl)((3-(trifluoromethyl)phenyl)carbamoyl)amide (IRBM-Z-2).

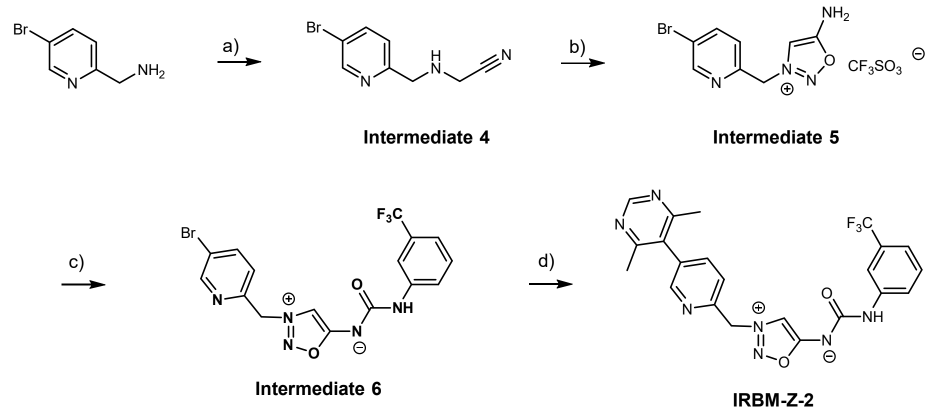

Supplementary Scheme 3 | Synthetic pathway for **IRBM-Z-2**: a) 2-bromoacetonitrile, DIPEA, MeCN, 60 °C, 4 h (98%); b) 1. *t*-butyl nitrite, THF, rt, 2 h; 2. trimethylsilyl trifluoromethanesulfonate, HCl (4N), dioxane, rt, 90 min (89% over two steps); c) 1-isocyanato-3-(trifluoromethyl)benzene, pyridine, MeCN, rt, 5 min (93%); d) 4,6-dimethyl-5-(4,4,5,5-tetramethyl-1,3,2-dioxaborolan-2-yl)pyrimidine, Pd(ddpf)Cl_2, K_3PO_4, H_2O/dioxane, 80 °C, 1 h (54%).

Step 1: 2-(((5-Bromopyridin-2-yl)methyl)amino)acetonitrile (Intermediate 4).

A suspension of (5-bromopyridin-2-yl)methanamine (8.4 g, 44.8 mmol) and DIPEA (23.4 mL, 134.4 mmol) in MeCN (89.6 mL) was treated with 2-bromoacetonitrile (3.4 mL, 49.3 mmol) and the reaction mixture was stirred at 60 °C for 4 h. Solvent was then removed under reduced pressure and the residue treated with water (25 mL) and extracted with EtOAc (3 x 75 mL). The collected organics were washed with brine, dried over Na_2SO_4 and evaporated under reduced pressure to get the title compound as a yellow oil (10.1 g, 98%). ^1H NMR (400 MHz, DMSO-d_6): δ 8.63 (d, J = 2.4 Hz, 1H), 8.03 (dd, J_1 = 8.4, J_2 = 2.4 Hz, 1H), 7.41 (d, 2H, J = 8.4 Hz, 1H), 3.84-3.82 (m, 2H), 3.69-3.65 (m, 2H), 3.25-2.18 (m, 1H); LCMS (ES^+): m/z calculated for $C_8H_8BrN_3$ 224.99, 226.99, found 226, 228 $[M+H]^+$; HPLC t_R = 1.26 min.

Step 2: 5-Amino-3-((5-bromopyridin-2-yl)methyl)-1,2,3-oxadiazol-3-ium trifluoromethyl-sulfonate (Intermediate 5).

A solution of 2-(((5-bromopyridin-2-yl)methyl)amino)acetonitrile (**Intermediate 4**) (10.1 g, 44.7 mmol) in THF (45.0 mL) was treated with *tert*-butyl nitrite (18.7 mL, 134.1 mmol). The solution was stirred at rt for 2 h. EtOAc was added to the mixture, the corresponding organic phase was washed with water and brine, dried over Na_2SO_4 and concentrated under reduced pressure to afford *N*-[(5-bromopyrimidin-2-yl)methyl]-*N*-(cyanomethyl)nitrous amide (11.3 g, 100%) as a brown residue, which was diluted with dioxane (55.4 mL) and treated with trimethylsilyl trifluoromethanesulfonate (29.5 g, 132.9 mmol) and HCl (4 M in dioxane, 22.2 mL, 88.6 mmol). The resulting reaction mixture was stirred at rt for 90 min, then quenched with water and concentrated under reduced pressure. The residue was dissolved in toluene, and the

excess solvent was removed under reduced pressure. This procedure was repeated twice. The title compound was obtained as an orange solid (21.9 g, 89%). ^{1}H NMR (400 MHz, DMSO-d_6): δ 9.51 (s, 2H), 8.76 (d, J = 1.9 Hz, 1H), 8.24 (dd, J_1 = 8.3, J_2 = 2.4 Hz, 1H), 8.07 (s, 1H), 7.67 (d, J = 8.3Hz, 1H), 6.04 (s, 2H); ^{19}F NMR (377 MHz, DMSO-d_6): δ -77.75 (s, 3F). LCMS (ES$^+$): m/z calculated for $C_8H_8BrN_4O^+$ 254.99, 256.99; found 254, 256 [M]$^+$; HPLC t_R = 0.57 min.

Step 3: (3-((5-Bromopyridin-2-yl)methyl)-1,2,3-oxadiazol-3-ium-5-yl)((3-(trifluoromethyl)-phenyl)carbamoyl)amide (Intermediate 6).

A suspension of 5-amino-3-((5-bromopyridin-2-yl)methyl)-1,2,3-oxadiazol-3-ium trifluorosulfonic (**Intermediate 5**, 10.1 g, 18.2 mmol) in anhydrous MeCN (52.0 mL) was treated at 0 °C with anhydrous pyridine (7.3 mL, 90.8 mmol) and 1-isocyanato-3-(trifluoromethyl)benzene (2.8 g, 20.0 mmol) and stirred at rt for 5 min before being quenched with water and extracted with EtOAc. The organic layer was washed with brine, dried over Na_2SO_4, filtered and concentrated in vacuo to give the crude product which was purified by chromatography on silica gel (eluent gradient from 10% to 100% EtOAc in petroleum ether) which afforded the title compound as a pale-yellow powder (7.44 g, 93%). ^{1}H NMR (400 MHz, DMSO-d_6): δ 9.73 (s, 1H), 8.76 (d, J = 1.9 Hz, 1H), 8.25-8.18 (m, 3H), 7.75-7.72 (m, 1H), 7.65 (d, J = 8.3 Hz, 1H), 7.45 (t, J = 7.8 Hz, 1H), 7.23 (d, J = 7.7 Hz, 1H), 5.94 (s, 2H); ^{19}F NMR (377 MHz, DMSO-d_6): δ -61.28 (s, 3F); LCMS (ES$^+$): m/z calculated for $C_{16}H_{11}BrF_3N_5O_2$ 441.00, 443.00; found 442, 444 [M+H]$^+$; HPLC t_R = 1.80 min.

Step 4: (3-((5-(4,6-Dimethylpyrimidin-5-yl)pyridin-2-yl)methyl)-1,2,3-oxadiazol-3-ium-5-yl)((3-(trifluoromethyl)phenyl)carbamoyl)amide (IRBM-Z-2).

A degassed suspension of (3-((5-bromopyridin-2-yl)methyl)-1,2,3-oxadiazol-3-ium-5-yl)((3-(trifluoromethyl)phenyl)carbamoyl)amide (**Intermediate 6**, 1.4 g, 3.17 mmol), 4,6-dimethyl-5-(4,4,5,5-tetramethyl-1,3,2-dioxaborolan-2-yl)pyrimidine (2.1 g, 6.33 mmol), Pd(dppf)Cl_2 (232 mg, 0.32 mmol) and K_3PO_4 (2.0 g, 9.5 mmol) in a mixture of dioxane (63 mL) and water (6 mL) was heated at 80 °C for 1 h before being cooled at rt, diluted with EtOAc and washed with water and brine, dried over Na_2SO_4 and evaporated in vacuo. The title compound was obtained after purification by RP-HPLC using water and MeCN as eluents (C18 column) to give the title compound as a white solid (458 mg, 31%). ^{1}H NMR (400 MHz, DMSO-d_6): δ 9.75 (br s, 1H), 8.93 (s, 1H), 8.59 (d, J = 1.8 Hz, 1H), 8.29 (s, 1H), 8.21 (s, 1H), 7.97 (dd, J_1 = 8.0 Hz, J_2 = 2.3 Hz, 1H), 7.79 (d, J = 8.0 Hz, 1H), 7.74 (br d, J = 8.5 Hz, 1H), 7.45 (t, J = 8.0 Hz, 1H), 7.23 (d, J = 7.9 Hz, 1H), 6.04 (s, 2H), 2.21 (s, 6H); ^{13}C NMR (101 MHz, DMSO-d_6): δ 173.2, 164.6, 159.5, 157.3, 151.6, 150.1, 142.4, 138.7, 132.7, 130.2, 130.0, 129.6, 126.2, 123.6, 121.8, 121.6, 117.8, 114.2, 106.2, 57.0, 23.3 (2C); ^{19}F NMR (377 MHz, DMSO-d_6) δ -61.28 (s, 3F); LCMS (ES$^+$): m/z calculated for $C_{22}H_{18}F_3N_7O_2$ 469.15; found 470 [M+H]$^+$; HPLC t_R = 1.52 min; HRMS (m/z): [M+H]$^+$ calculated for $C_{22}H_{18}F_3N_7O_2$, 470.15468; found 470.15485.

^{1}H NMR, ^{19}F NMR and ^{13}C NMR spectra, UPLC-MS and HRMS of compounds IRBM-Z-1 and IRBM-Z-2

UPLC-MS of IRBM-Z-1 Method 10-90.

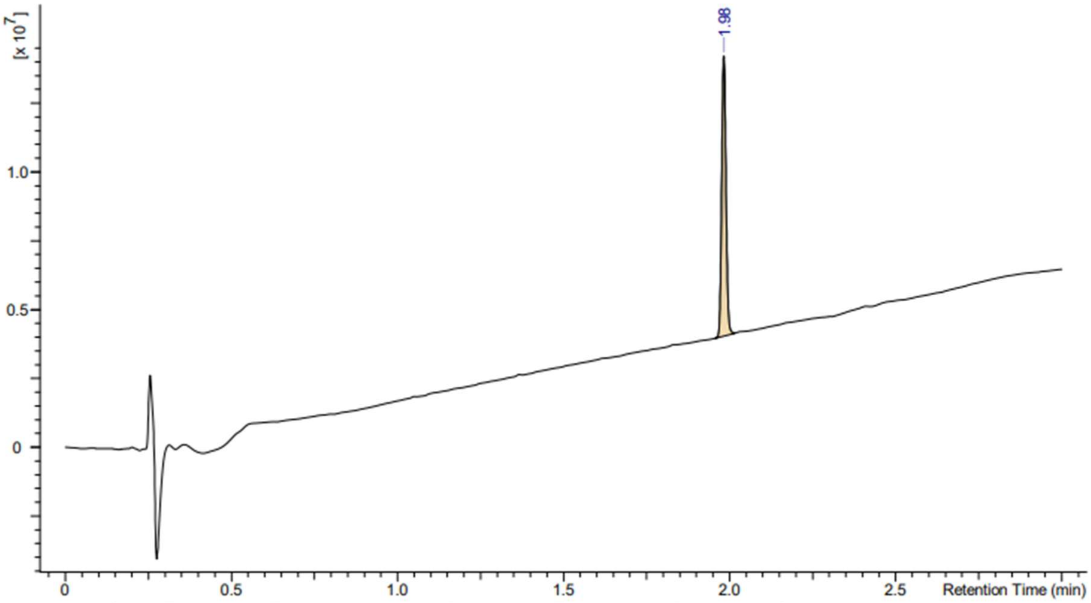

No.	tR	Peak Area (Y units*ms)	Area Percent	Height (counts)	Width
1	1.98	8933776875	100.0	-	0.023

Gradient Table

Time (min)	Flow Rate	%A	%B	Curve
Initial	0.500	90.0	10.0	Initial
0.10	0.500	90.0	10.0	6
2.60	0.500	0.0	100.0	6
2.90	0.500	0.0	100.0	6
3.00	0.500	90.0	10.0	6

UPLC gradient: A = H_20 + 0.1% HCO_2H; B = MeCN + 0.1% HCO_2H

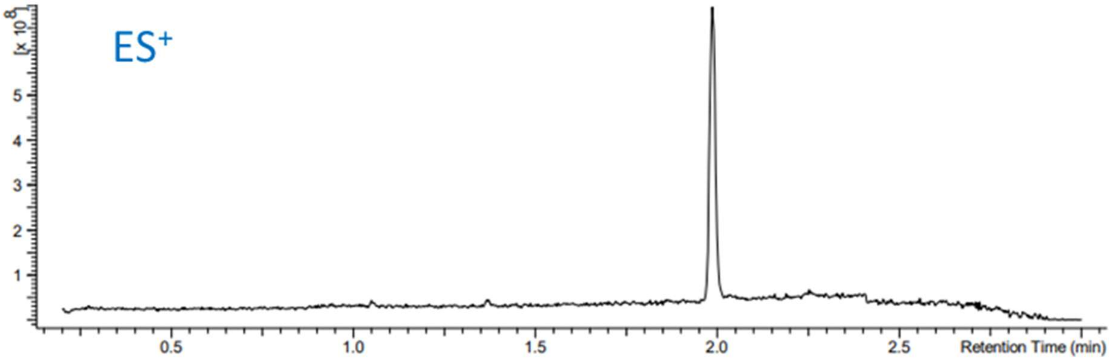

COMBINED MASS SPECTRA

Retention Time 1.983 **Ion Mode** ES+ **Spectrum Type** MS **Combine** <556-574>

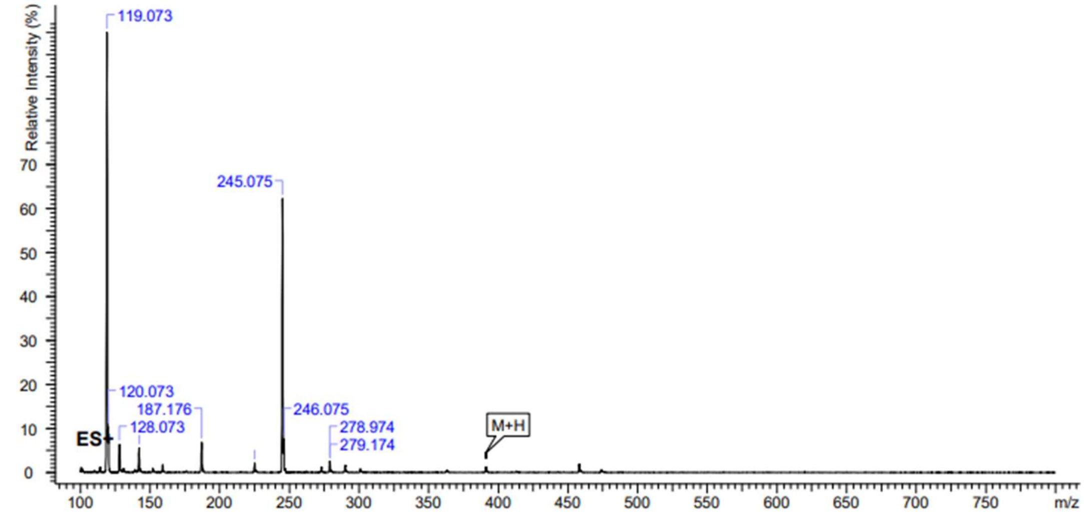

UPLC of IRBM-Z-1 Method 10-70.

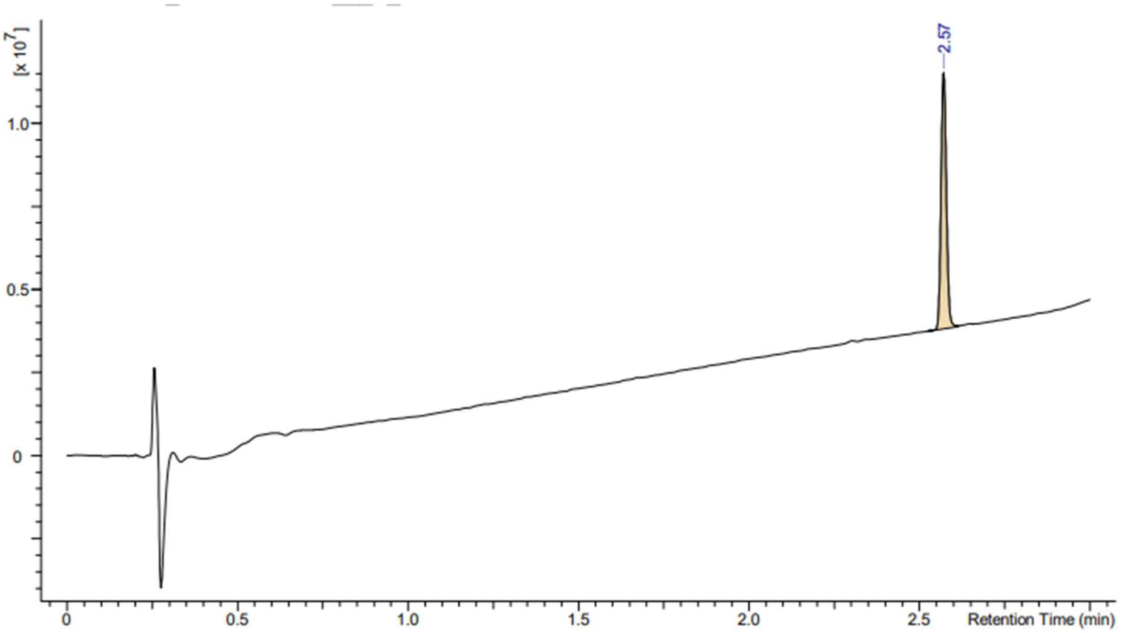

No.	tR	Peak Area (Y units*ms)	Area Percent	Height (counts)	Width
1	2.57	8198349375	100.0	-	0.028

Gradient Table

Time (min)	Flow Rate	%A	%B	Curve
Initial	0.500	90.0	10.0	Initial
0.10	0.500	90.0	10.0	6
2.60	0.500	30.0	70.0	6
2.90	0.500	0.0	100.0	6
3.00	0.500	90.0	10.0	6

UPLC gradient: A = H_20 + 0.1% HCO_2H; B = MeCN + 0.1% HCO_2H

UPLC of IRBM-Z-1 Method 40-80.

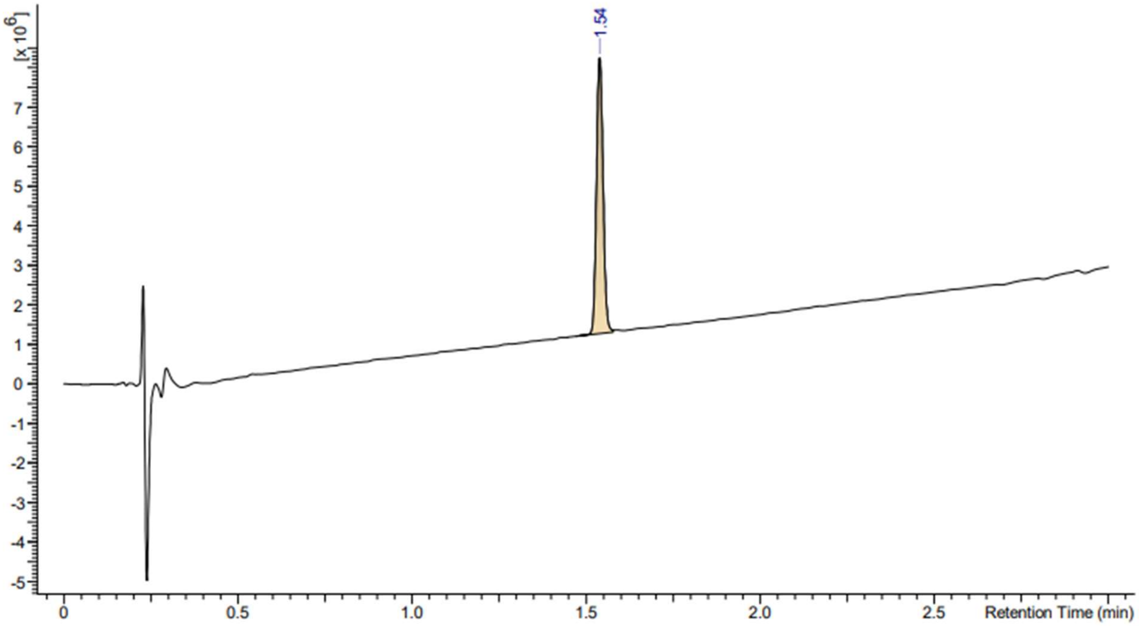

No.	tR	Peak Area (Y units*ms)	Area Percent	Height (counts)	Width
1	1.54	9109654688	100.0	-	0.035

Gradient Table

Time (min)	Flow Rate	%A	%B	Curve
Initial	0.500	60.0	40.0	Initial
0.10	0.500	60.0	40.0	6
2.60	0.500	20.0	80.0	6
2.90	0.500	0.0	100.0	6
3.00	0.500	90.0	10.0	6

UPLC gradient: A = H_20 + 0.1% HCO_2H; B = MeCN + 0.1% HCO_2H

^{1}H NMR spectrum of compound IRBM-Z-1.

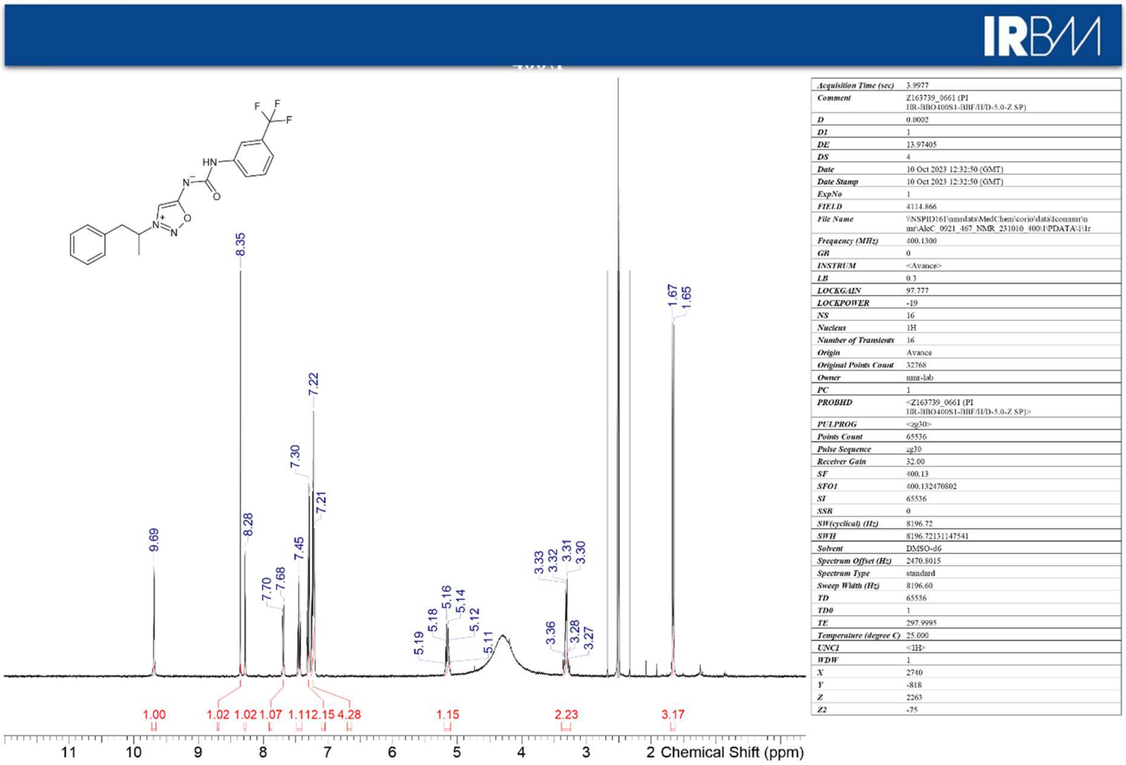

^{13}C NMR spectrum of compound IRBM-Z-1.

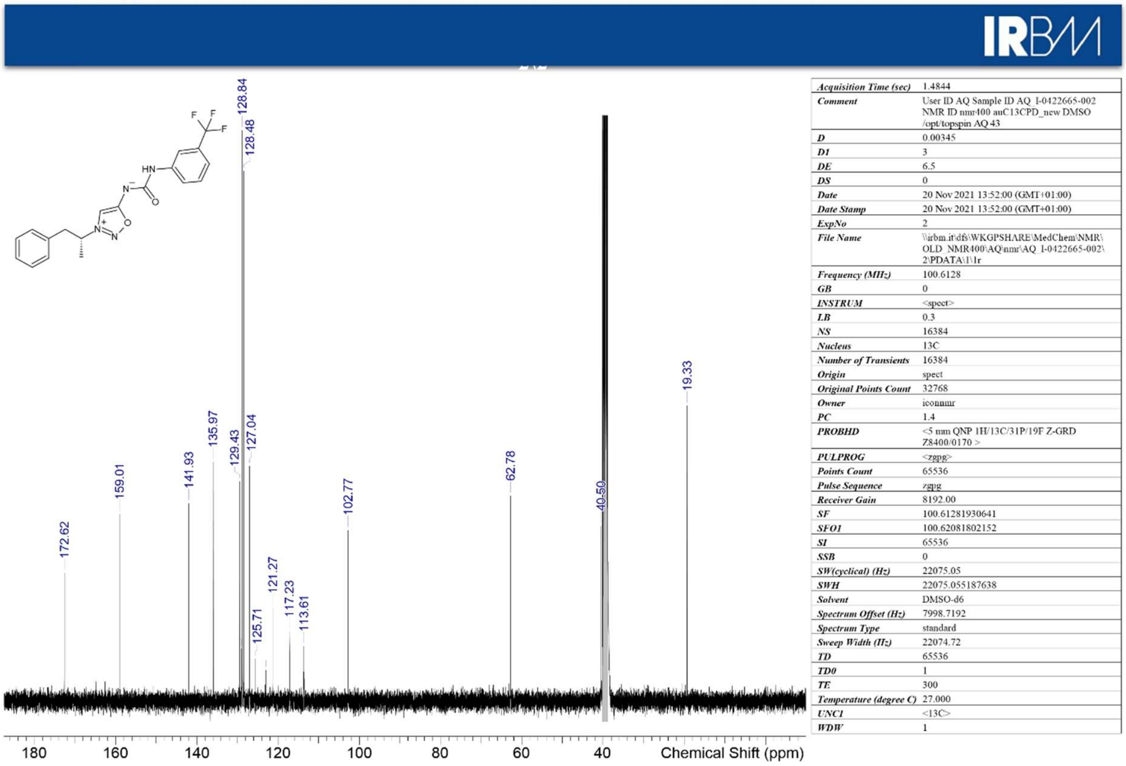

^{19}F NMR spectrum of compound IRBM-Z-1.

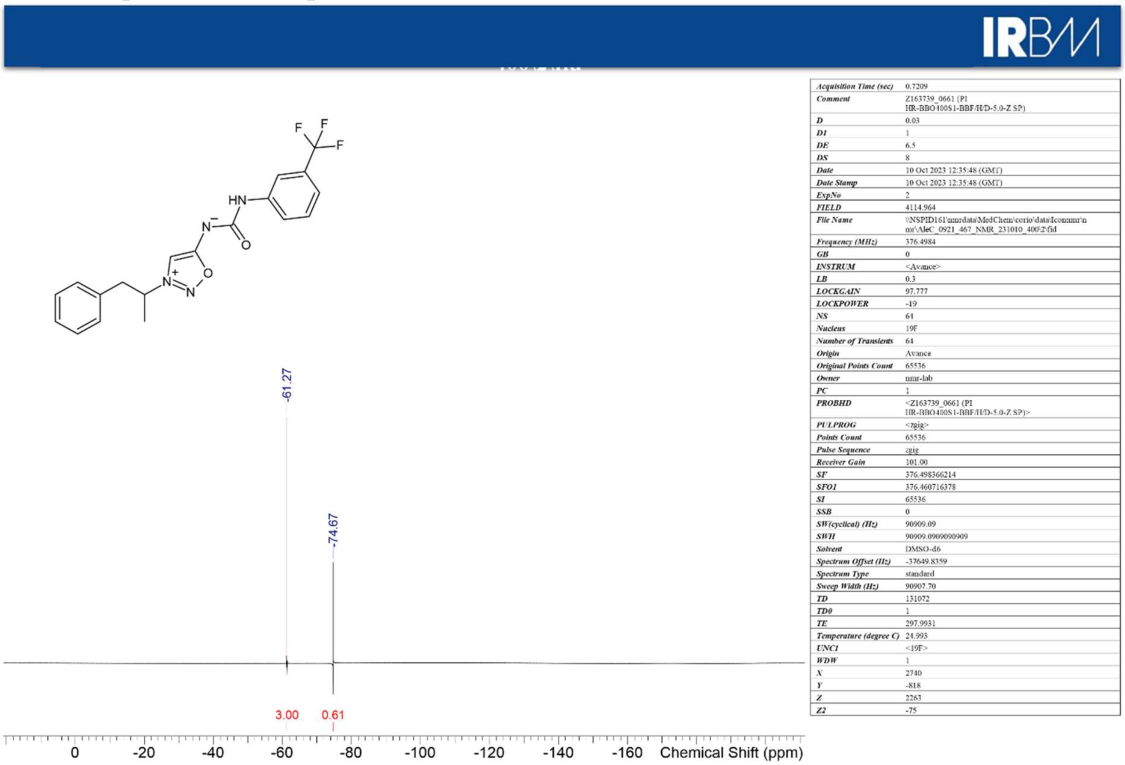

Acquisition Time (sec)	0.7209
Comment	Z163739_0661 (PI HR-BBO400S1-BBF/H/D-5.0-Z SP)
D	0.03
D1	1
DE	6.5
DS	8
Date	10 Oct 2023 12:35:48 (GMT)
Date Stamp	10 Oct 2023 12:35:48 (GMT)
ExpNo	2
FIELD	4114.964
File Name	\\NSPID161\nmrdata\MedChem\corio\data\Iconnmr\nmr\AleC_0921_467_NMR_231010_400\2\fid
Frequency (MHz)	376.4984
GB	0
INSTRUM	<Avance>
LB	0.3
LOCKGAIN	97.777
LOCKPOWER	-19
NS	64
Nucleus	19F
Number of Transients	64
Origin	Avance
Original Points Count	65536
Owner	nmr-lab
PC	1
PROBHD	<Z163739_0661 (PI HR-BBO400S1-BBF/H/D-5.0-Z SP)>
PULPROG	<zgig>
Points Count	65536
Pulse Sequence	zgig
Receiver Gain	101.00
SF	376.498366214
SFO1	376.460716378
SI	65536
SSB	0
SW(cyclical) (Hz)	90909.09
SWH	90909.0909090909
Solvent	DMSO-d6
Spectrum Offset (Hz)	-37649.8359
Spectrum Type	standard
Sweep Width (Hz)	90907.70
TD	131072
TD0	1
TE	297.9931
Temperature (degree C)	24.993
UNC1	<19F>
WDW	1
X	2740
Y	-818
Z	2263
Z2	-75

HRMS of IRBM-Z-1.

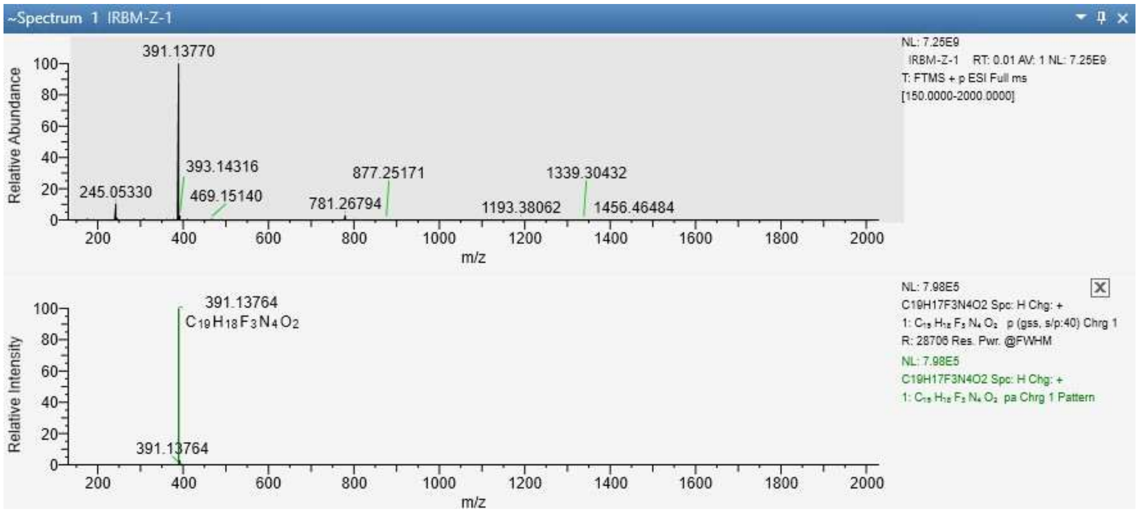

UPLC-MS of IRBM-Z-2 Method 10-90.

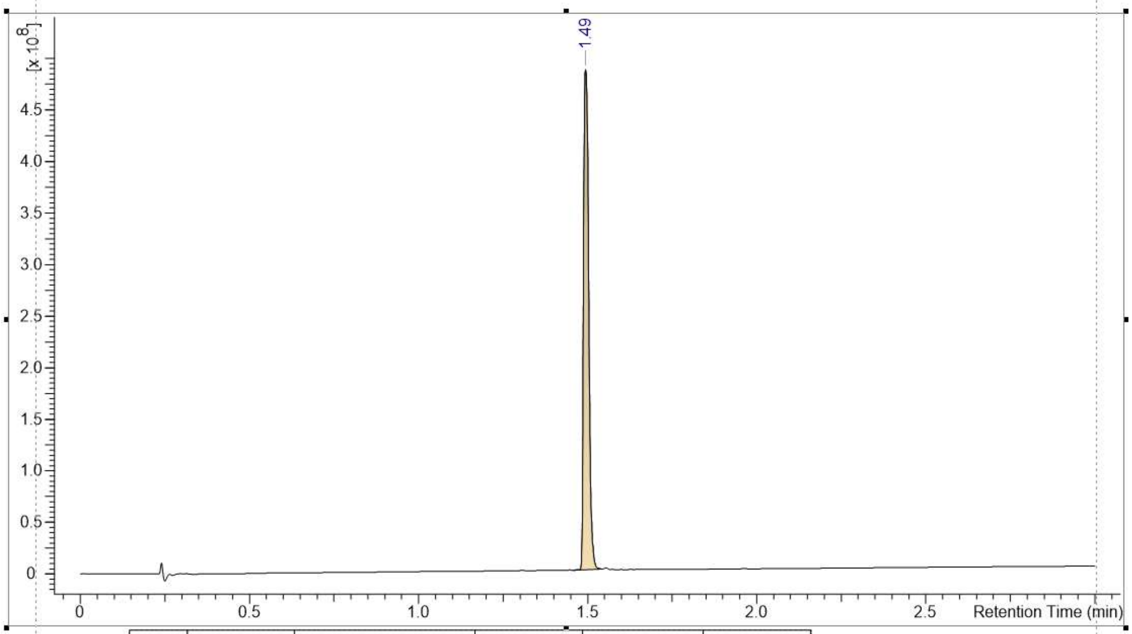

No.	tR	Peak Area (Y units*ms)	Area Percent	Height (counts)	Width
1	1.49	506921040000	100.0	-	0.028

Gradient Table

Time (min)	Flow Rate	%A	%B	Curve
Initial	0.500	90.0	10.0	Initial
0.10	0.500	90.0	10.0	6
2.60	0.500	0.0	100.0	6
2.90	0.500	0.0	100.0	6
3.00	0.500	90.0	10.0	6

UPLC gradient: A = H_20 + 0.1% HCO_2H; B = MeCN + 0.1% HCO_2H

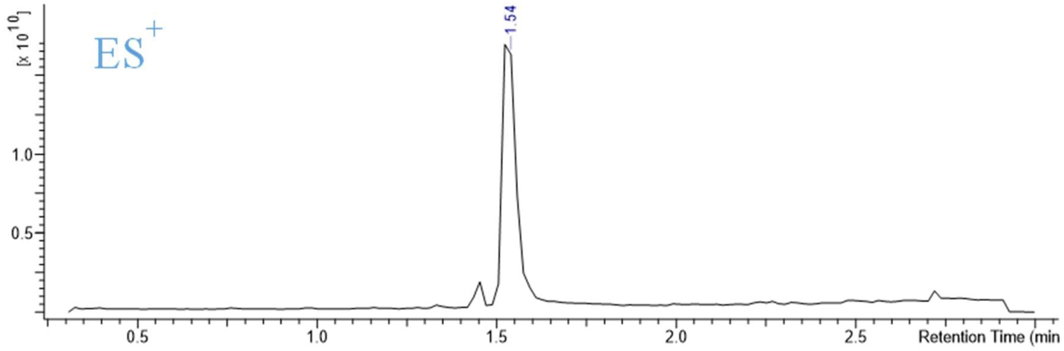
ES+
[x 10^10]
1.54
1.0
0.5
0.5
1.0
1.5
2.0
2.5
Retention Time (min

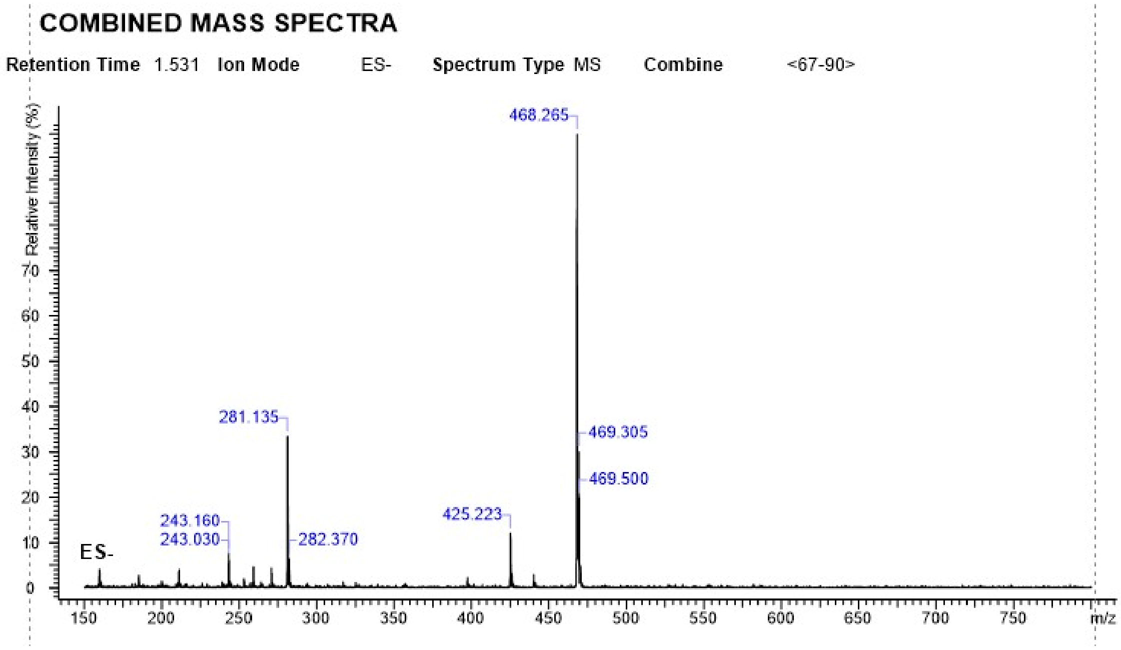
COMBINED MASS SPECTRA
Retention Time 1.531 Ion Mode ES- Spectrum Type MS Combine <67-90>
Relative Intensity (%)
468.265
469.305
469.500
281.135
282.370
243.160
243.030
425.223
ES-
m/z

UPLC of IRBM-Z-2 Method 10-60.

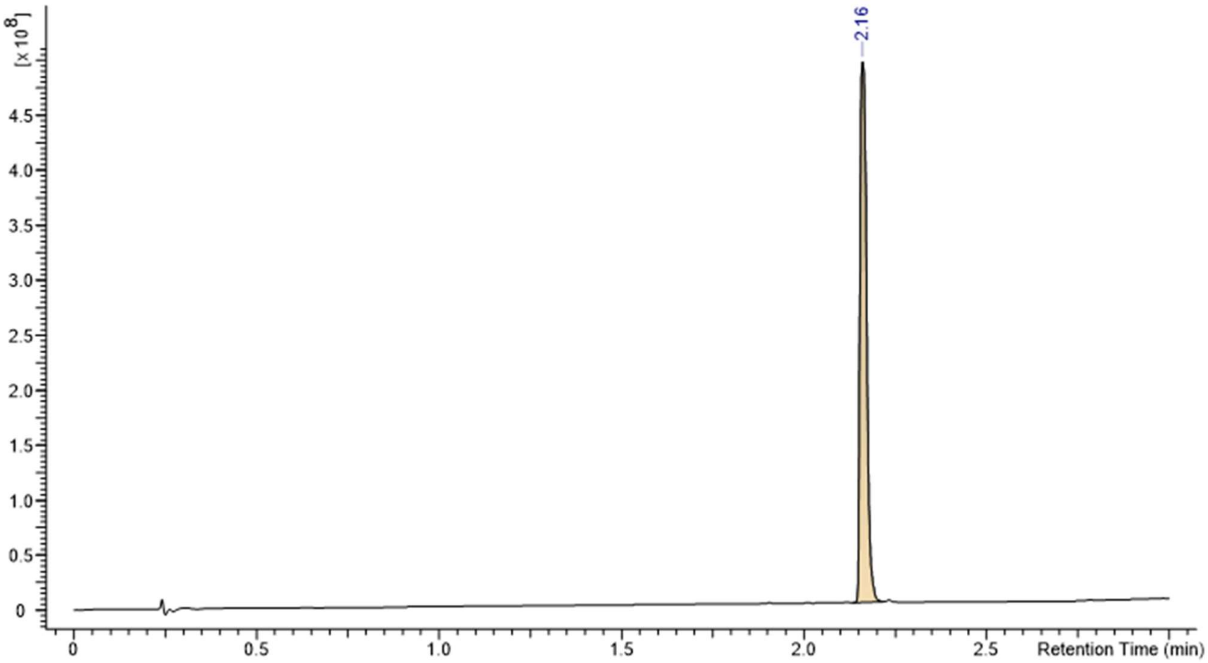

No.	tR	Peak Area (Y units*ms)	Area Percent	Height (counts)	Width
1	2.16	614122380000	100.0	-	0.034

Gradient Table

Time (min)	Flow Rate	%A	%B	Curve
Initial	0.500	90.0	10.0	Initial
0.50	0.500	90.0	10.0	6
2.40	0.500	40.0	60.0	6
2.70	0.500	0.0	100.0	6
2.90	0.500	0.0	100.0	6
3.00	0.500	100.0	0.0	6

UPLC gradient: A = H_20 + 0.1% HCO_2H; B = MeCN + 0.1% HCO_2H

UPLC of IRBM-Z-2 Method 40-80.

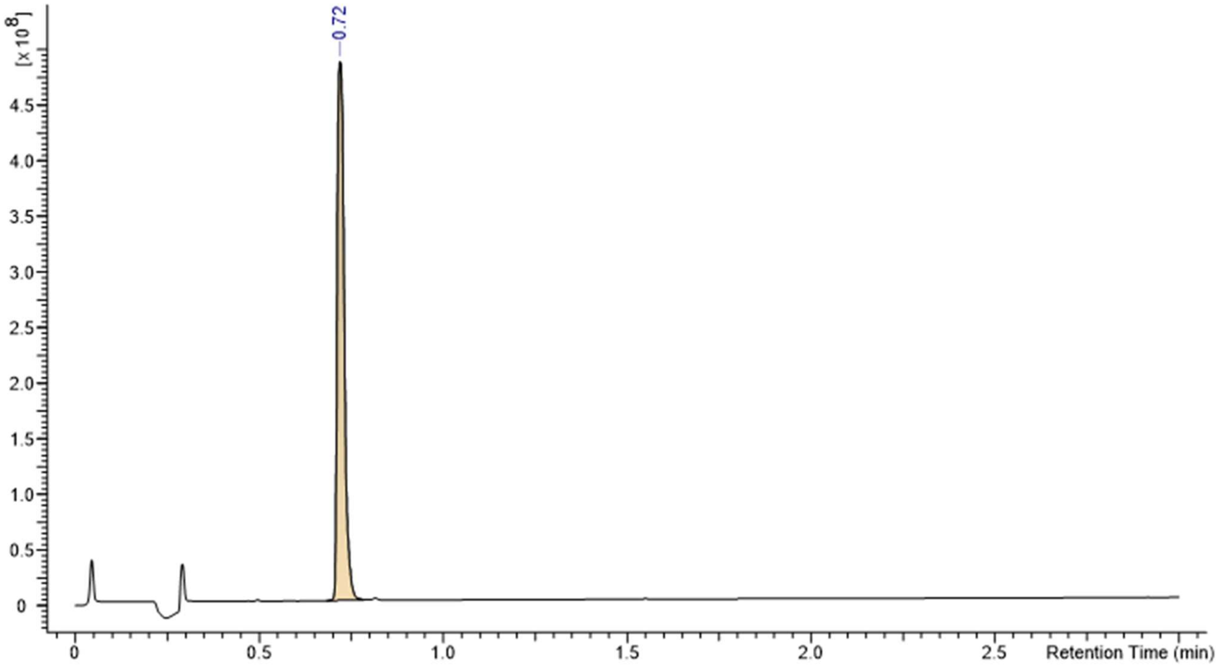

No.	tR	Peak Area (Y units*ms)	Area Percent	Height (counts)	Width
1	0.72	640328460000	100.0	-	0.035

Gradient Table

Time (min)	Flow Rate	%A	%B	Curve
Initial	0.500	60.0	40.0	Initial
0.10	0.500	60.0	40.0	6
2.60	0.500	20.0	80.0	6
2.90	0.500	0.0	100.0	6
3.00	0.500	90.0	10.0	6

UPLC gradient: A = H_20 + 0.1% HCO_2H; B = MeCN + 0.1% HCO_2H

^{1}H NMR spectrum of compound IRBM-Z-2.

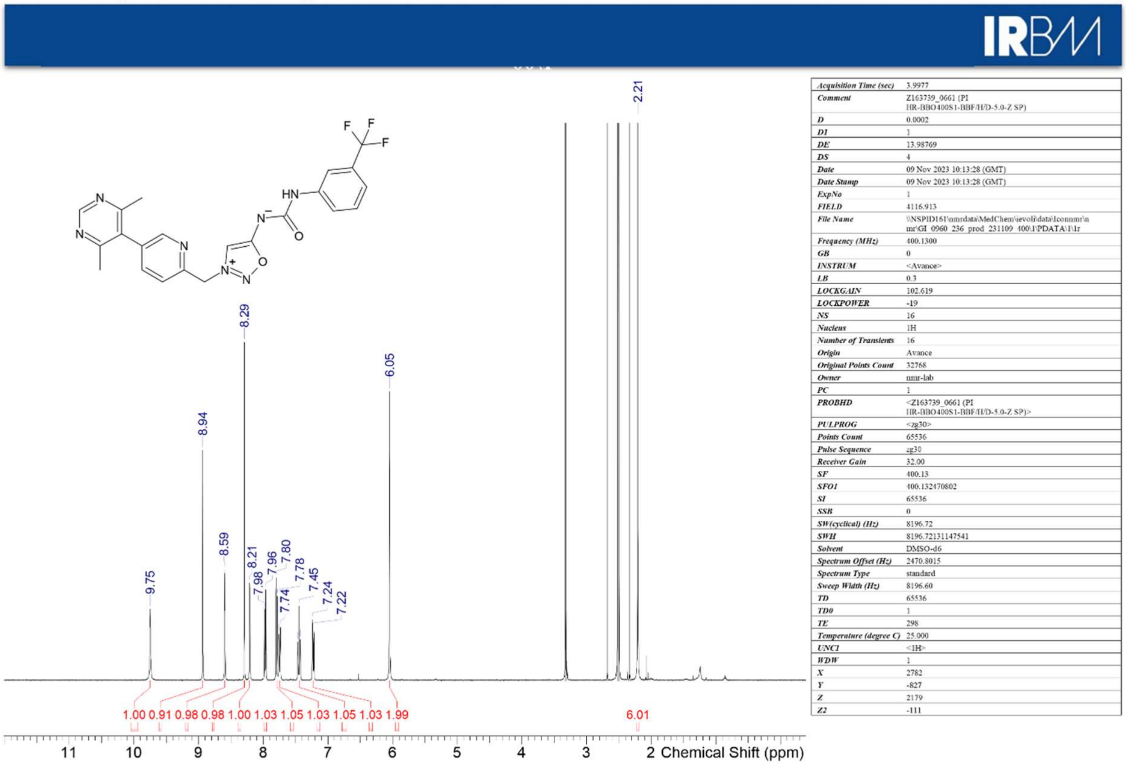

^{13}C NMR spectrum of compound IRBM-Z-2.

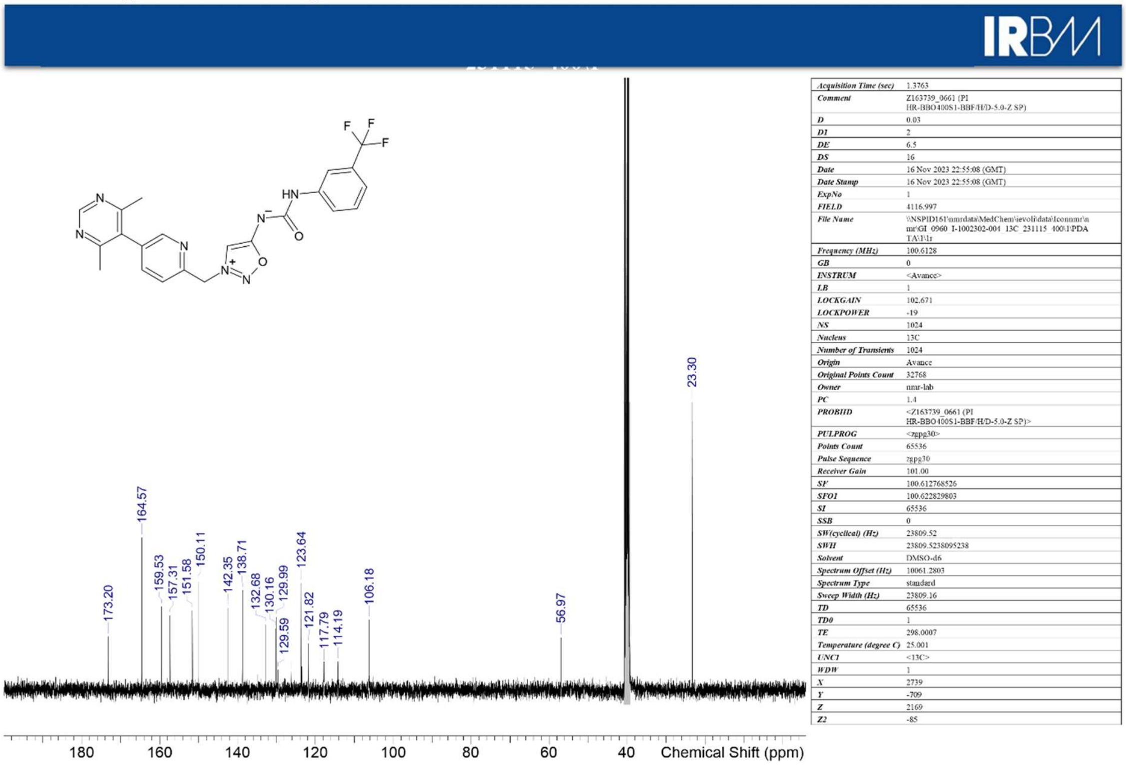

^{19}F NMR spectrum of compound IRBM-Z-2.

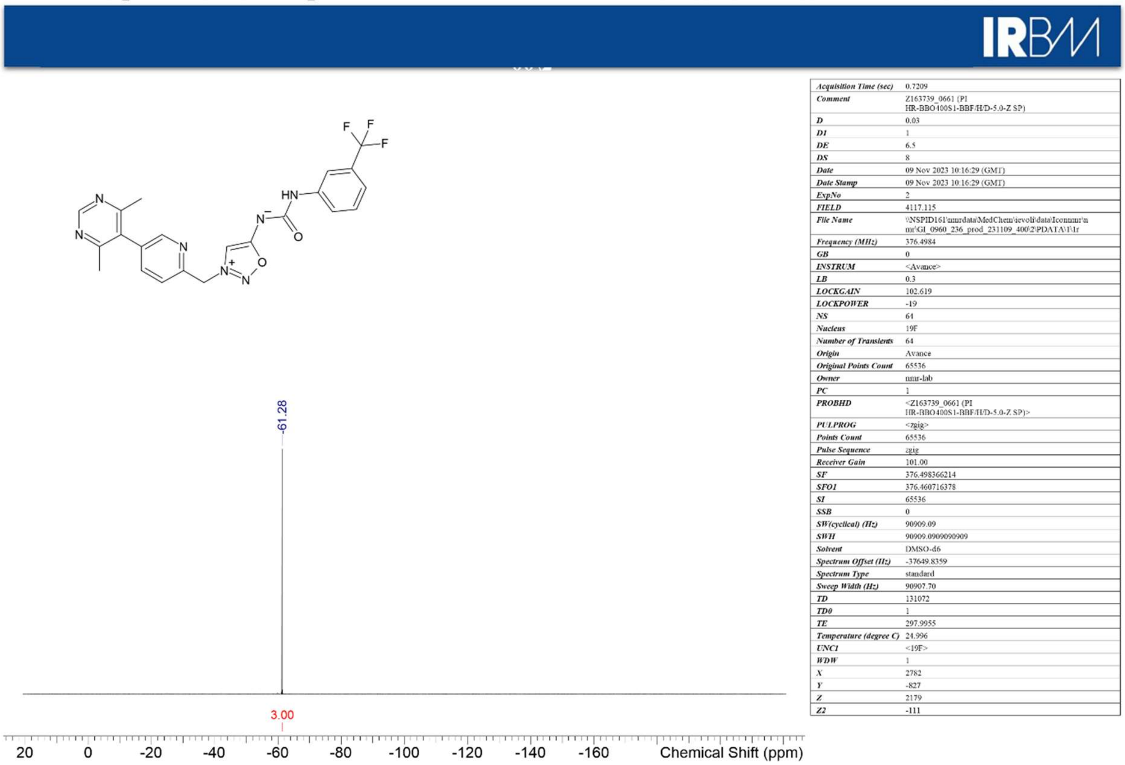

Acquisition Time (sec)	0.7209
Comment	Z163739_0661 (PI HR-BBO400S1-BBF/H/D-5.0-Z SP)
D	0.03
D1	1
DE	6.5
DS	8
Date	09 Nov 2023 10:16:29 (GMT)
Date Stamp	09 Nov 2023 10:16:29 (GMT)
ExpNo	2
FIELD	4117.115
File Name	\\NSPID161\nmrdata\MedChem\ievoli\data\Iconnmr\nmr\GI_0960_236_prod_231109_400\2\PDATA\1\1r
Frequency (MHz)	376.4984
GB	0
INSTRUM	<Avance>
LB	0.3
LOCKGAIN	102.619
LOCKPOWER	-19
NS	64
Nucleus	19F
Number of Transients	64
Origin	Avance
Original Points Count	65536
Owner	nmr-lab
PC	1
PROBHD	<Z163739_0661 (PI HR-BBO400S1-BBF/H/D-5.0-Z SP)>
PULPROG	<zgig>
Points Count	65536
Pulse Sequence	zgig
Receiver Gain	101.00
SF	376.498366214
SFO1	376.460716378
SI	65536
SSB	0
SW(cyclical) (Hz)	90909.09
SWH	90909.0909090909
Solvent	DMSO-d6
Spectrum Offset (Hz)	-37649.8359
Spectrum Type	standard
Sweep Width (Hz)	90907.70
TD	131072
TD0	1
TE	297.9955
Temperature (degree C)	24.996
UNC1	<19F>
WDW	1
X	2782
Y	-827
Z	2179
Z2	-111

HRMS of IRBM-Z-2.

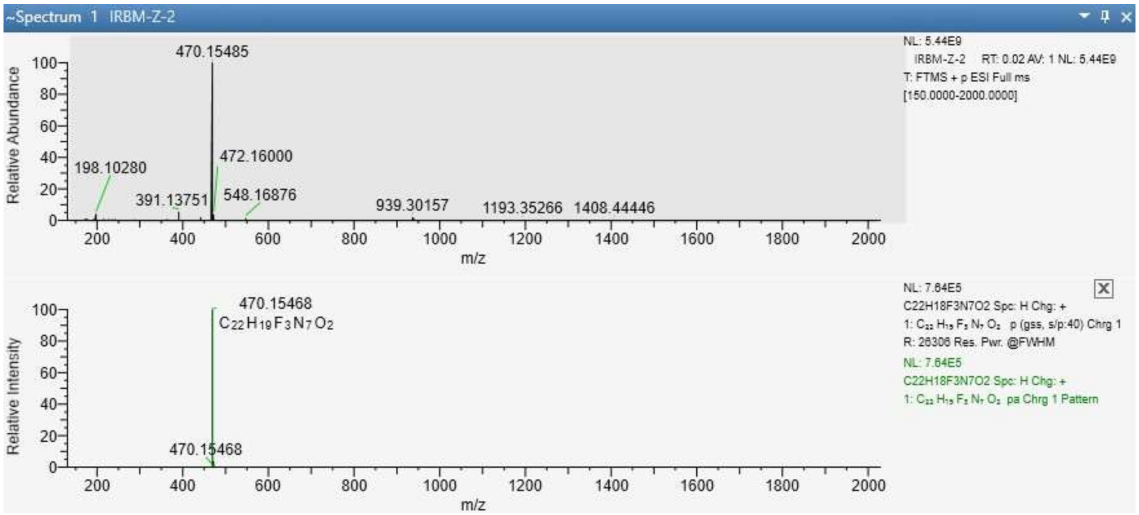

Supplementary References

1. Capuzzi, S. J. et al. Phantom PAINS: Problems with the Utility of Alerts for Pan-Assay Interference Compounds. *J. Chem. Inf. Model.* **57**, 417-427 (2017).
2. Baell, J. B. et al. Screening-based translation of public research encounters painful problems. *ACS Med. Chem. Lett.* **6**, 229-234 (2015).
3. Baell, J. B. et al. Chemistry: Chemical con artists foil drug discovery. *Nature* **513**, 481-483 (2014).
4. Johnston, P. A. et al. Redox cycling compounds generate H_2O_2 in HTS buffers containing strong reducing reagents-real hits or promiscuous artifacts? *Curr. Opin. Chem. Biol.* **15**, 174-182 (2011).
5. Soares, K. M. et al. Profiling the NIH Small Molecule Repository for compounds that generate H_2O_2 by redox cycling in reducing environments. *Assay Drug Dev. Technol.* **8**, 152-174 (2010).
6. Yao, Y. et al. Discovery X-ray Crystallography and Antiviral Activity of Allosteric Inhibitors of Flavivirus NS2B-NS3 Protease. *J. Am. Chem. Soc.* **141**, 6852-6836 (2019).
7. Millies, B. et al. Proline-Based Allosteric inhibitors of Zika and Dengue Virus NS2B/NS3 Proteases. *J. Med. Chem.* **62**, 11359-11382 (2019).
8. Coluccia, A. et al. Discovery of Zika Virus NS2B/NS3 Inhibitors That Prevent Mice from Life-Threatening Infection and Brain Damage. *ACS Med. Chem. Lett.* **11**, 1869-1874 (2020).
9. Erbel, P. A. et al. Structural basis for the activation of flaviviral NS3 proteases from dengue and West Nile virus. *Nat. Struct. Mol. Biol.* **13**, 372-373 (2006).
